# Using microartifacts to infer Middle Pleistocene lifeways at Schöningen, Germany

**DOI:** 10.1038/s41598-022-24769-3

**Published:** 2022-12-15

**Authors:** Flavia Venditti, Bárbara Rodríguez-Álvarez, Jordi Serangeli, Stella Nunziante Cesaro, Rudolf Walter, Nicholas J. Conard

**Affiliations:** 1 Department of Early Prehistory and Quaternary Ecology, University of Tübingen, Schloß Hohentübingen, Burgsteige 11, 72070 Tübingen, Germany; 2 Senckenberg Centre for Human Evolution and Palaeoenvironment, University of Tübingen, Paläon 1, 38364 Schöningen, Germany; 3 Scientific Methodologies Applied to Cultural Heritage (SMATCH), Rome, Italy; 4 Senckenberg Centre for Human Evolution and Palaeoenvironment, University of Tübingen, Rümelinstrasse 23, 72070 Tübingen, Germany

**Keywords:** Archaeology, Cultural evolution

## Abstract

While archeologists usually favor the study of large and diagnostic lithic artifacts, this study illustrates the invaluable contribution of lithic microartifacts for interpreting hominin lifeways. Across a 64 m^2^ area of the Middle Pleistocene lakeshore site of Schöningen 13 II-3 in Northern Germany, we recovered a total of 57 small and micro flint artifacts, four small debris pieces, three natural fragments and three bone retouchers in close association with the skeleton of an extinct Eurasian straight-tusked elephant (*Palaeoloxodon antiquus*). This area lacks the type of formal knapped stone tools that would normally constitute the focus of archeological interpretations. By adopting a holistic approach, including morpho-technical analysis, experimental archeology, and use-wear and residue analyses, we demonstrate that these small and microartifacts are resharpening flakes that tell the story of the site. Fifteen resharpening flakes preserve microwear traces of processing wood. Microscopic residues of wood adhered to the former working edges of the tools corroborate this observation. Additionally, hominins used a sharp-edged, natural fragment of flint to process fresh animal tissue, which likely originates from the butchery of the elephant. These results provide unique, 300,000-year-old evidence for the functionally interconnected use of lithic, osseous and wood technologies. Furthermore, we document *in-situ* transformations of stone tools and the presence of both curational and expedient behaviors, thereby demonstrating the temporal depth of hominin activities at the lakeshore where the elephant died, and in the broader landscape as a whole.

## Introduction

Material culture is a fundamental source of information for understanding human behavioral diversity in the past^1,2^. In that regard, stone artifacts have always played a major role in the understanding of the cultural aspects of early hominins, being the most abundant finds recovered from Paleolithic sites.

Traditionally, archeologists have favored the study of large and diagnostic lithic artifacts, at the expense of the smaller fraction (Supplementary Information). Researchers assumed that the smaller an artifact is, the less information it could provide^3^. Moreover, it was assumed that microartifacts simply mirror the same information as larger artifacts^4^. On the contrary, the feasibility and the interpretative potential of microartifact analysis has been demonstrated by multiple studies within the framework of site formation processes (e.g.,^5–12^). Scholars like Schiffer^7^ and McKeller^13^ noted that microartifacts are more prone to stay where they are dropped, while macroartifacts are often removed from their original location of use, thus helping archaeologists interpret primary activity areas and site formation. This principle, theorized as the “McKeller Hypothesis”^13^ shows that microartifacts may provide different information about the archaeological record than macroartifacts do. Furthermore, microartifacts are abundant cultural remains in the archaeological record and they are occasionally better preserved than their larger counterparts. For those reasons, Dunnel and Stein^3^ advocated the need to supplement and complement macroartifact investigation with microartifact analysis.

Although the importance of microartifacts in archaeology has been recognized and recently discussed (e.g.,^3,4,14–19^), collection is not always systematic and analysis is not fully integrated into standard archaeological practice. It follows that microartifacts are still rarely technologically investigated, and their functional aspects are hardly addressed (see^20,21^). This is mostly due to a series of factors including (1) the need for careful recovery techniques in the field, (2) the tedious work of classification and quantification, (3) the tiny size of the artifacts, often requiring the use of microscopy, (4) the challenge of analysing minute surfaces under the lens of microscopes.

Starting from these premises, we show here the invaluable contribution of microartifact analysis for interpreting the lifeways, subsistence and technology of Middle Pleistocene hominins at Schöningen (henceforth Schö).

The lithic assemblage of the 64 m^2^ studied area at Schö 13 II-3 is almost entirely composed of tiny small and microflakes recovered in close association with an almost complete skeleton of an extinct Eurasian straight-tusked elephant (Supplementary Information and Supplementary Fig. 1). Three natural flint fragments and 4 pieces of angular debris complement the sample. Our aim here is fourfold: (1) characterize the attributes of the knapped products; (2) test the potential of microartifacts (i.e., resharpening flakes) for reconstructing site function; (3) determine the use of tools prior to resharpening; and (4) reconstruct technological and functional activities performed at the location of the elephant.

In doing so, we applied a holistic approach integrating different techniques in a single, comprehensive study. We combined a spatial investigation with data from a classic morphological and technological analysis coupled with microwear analysis integrated with optical, elemental and chemical characterization of residues. Laboratory analyses are complemented by actualistic experiments aimed at observing resharpening debitage and recording the development and distribution of wear traces and residues on the small and microflakes (see Supplementary Information).

The results show that several of the analysed resharpening flakes from Schö 13 II-3 exhibit traces of processing wood on their striking platforms, which correspond to the former working edges of tools. Microscopic residues compatible with woody tissues adhering on these butts and sticking to the dorsal proximal retouch scars corroborate this observation. Besides woodworking, one natural shard shows evidence of use compatible with a cutting activity on fresh animal tissues.

These results provide evidence for curational behavior at the elephant area while documenting the production and/or maintenance of wooden tools. Moreover, they show the coexistence of curated and expedient behaviors within the technological organization of the Schöningen hominins ca. 300,000 years ago. From a methodological perspective, this study proves that use-wear and residue analyses of microartifacts provide valuable information concerning tool use and technological activities.

## Results

### Morpho-technical and spatial analysis

**Figure 1:**
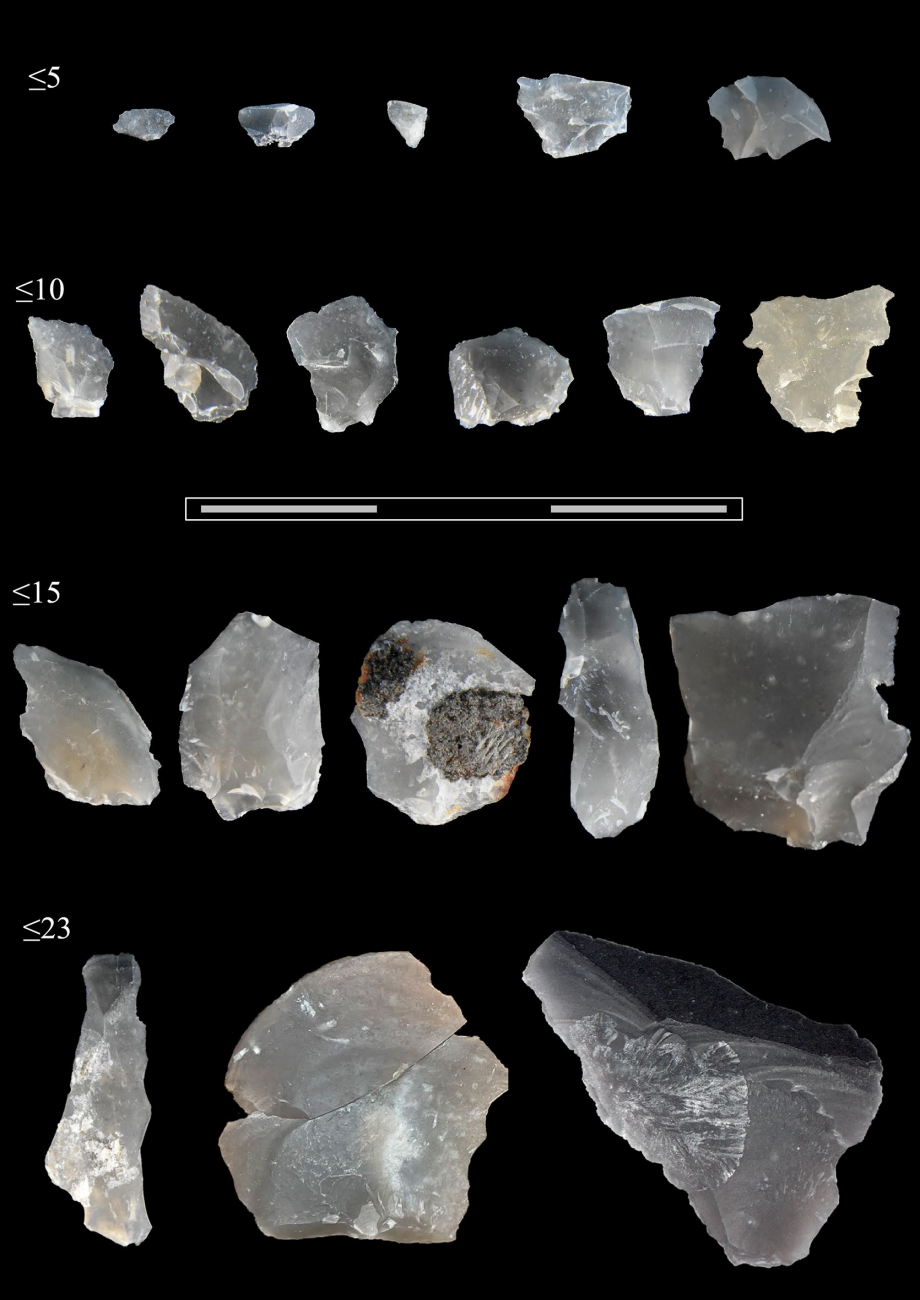
Overview of the resharpening flakes from Schöningen 13 II-3. Complete flakes are grouped attending to the technological dimension and according to the following ranges identified in the assemblage: ≤ 5 mm (N = 8); ≤ 10 mm (N = 21); ≤ 15 mm (N = 12); ≤ 23 mm (N = 3).

The raw material of all lithic remains recovered from the studied area is flint. Except for four pieces of debris and a frost fragment directly used without previous transformation (ID 29967), the remaining artifacts consist of 57 unretouched flakes (Fig. 1). Therefore, the analysed morphotechnical characteristics correspond to attributes on the butt, dorsal and ventral sides of these pieces (except when their small size hampered analysis). Additionally, together with the 77.2% of complete flakes, some attributes were considered for analysis on incomplete pieces if they were not hindered by fractures.

Furthermore, among the flakes, we identified one conjoin (ID 29675; ID 30251), which has been analysed as a single flake, and a refit of two flakes (ID 29716; ID 29817). According to its morphology, size and color, a third piece (ID 29377) without a direct refit may have been removed within the same group of blows (Supplementary Fig. 1).

**Figure 2:**
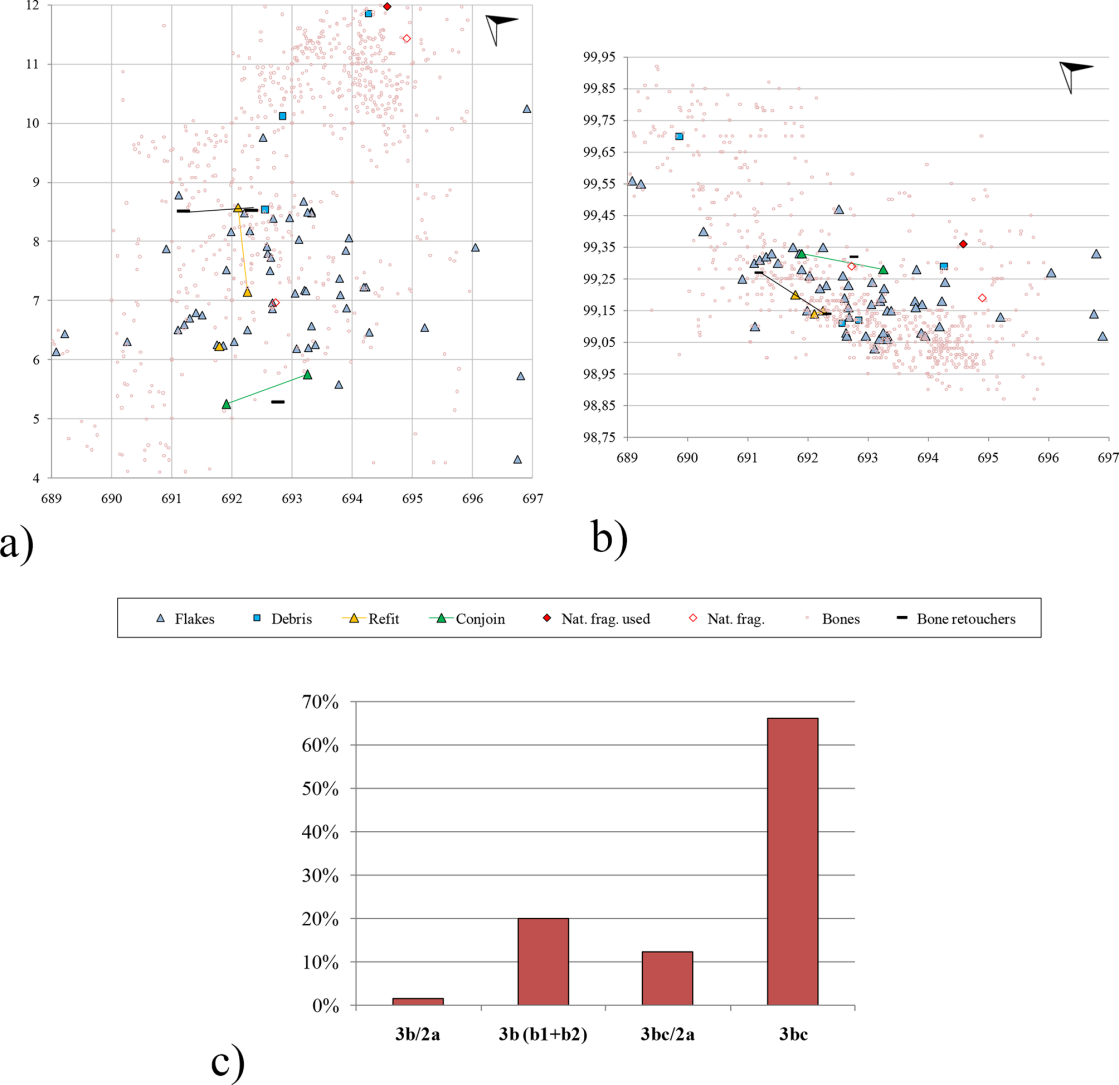
Spatial distribution of finds from the elephant area at Schöningen 13 II-3. State of research in August 2022. (**a,b**) horizontal (x–y axis) and vertical (x–z axis) distribution of the lithic and faunal remains; (**c**) distribution of the lithic record among the layers.

Concerning the spatial distribution of the finds, the lithic remains found around the elephant were recovered from a total of 25 square meters, although half of the squares only contained a single artifact. The maximum collected from a single square was 8 microflakes (six of them were identified during sieving). The pieces are vertically distributed across 70 cm, concentrating in an area of 40 cm. The two small flakes that refit were found around 1.5 m horizontally from one another (Supplementary Fig. 1, picture in the middle) and just 1 cm vertically. As for the conjoin, the two pieces were located also around 1.5 m horizontally and 5 cm vertically (Fig. 2a). The natural fragment with use traces was lying on layer 3b, which is the second most represented (sub-layers 3b1 and 3b2 were grouped together) (Fig. 2b). The majority of the lithic assemblage was found in layer 3bc, which includes most of the flakes and some of the debris (Fig. 2c). The conjoin and the refit were also found in this layer.

It is worth mentioning that the spatial distribution (close proximity of the refits and conjoins) proves that the knapped products were not transported by water over a great distance, meaning their location reflects the original area of deposition.

#### Results on the flakes

The flaking length of complete artifacts is distributed between 2 and 23 mm, and these dimensions do not change when incomplete products are included. The largest width of the flakes is recorded on a broken product that, if complete, would not exceed 3 cm, since the missing part would not increase its flaking width (Supplementary Fig. 2). Concerning the average of the technological dimension (length and width), there is no flake larger or wider than 1 cm. The artifacts are also very thin; the thickness of most pieces measures under 2 mm (70.2%). As expected, the lowest values correspond to the weight with a mean of 0.2 g and a maximum of 1.9 g, which would not change even if the broken flakes were complete (Supplementary Table 1).

A close look at the technological length of complete flakes reveals that the majority of the sample corresponds to microflakes, especially those ranging between 5 and 10 mm. Even though chips and small flakes are equally represented, 91% of the debitage does not exceed 15 mm. As the length ranges increase, so does the mean of their width, but with little difference and never exceeding the average length (and Supplementary Table 2). The elongation index of 1.2 (S.D. = 0.5) also confirms that most flakes are longer than they are wide (Supplementary Fig. 3).

The analyses of the attributes show that, on the ventral sides, lips are present on most products. The presence of bulbs was recorded on almost half of the lithic artifacts. Most of these ventral faces follow concave as well as some straight delineations. The majority of flakes carry feathered terminations (Supplementary Table 3).

The identification of the type of butt has been partly hampered by their very small size, and sometimes it was not possible to identify platforms or lineal ends. Nonetheless, these two are the most common type of butts in the assemblage. All flakes have unifaceted butts without cortex, and the delineation could only be recorded on a small part of the sample, showing a tendency of straight surfaces (Supplementary Table 4).

Considering the small dimensions of the studied sample it is not surprising that, with the exception of one of the biggest flakes, the butt width does not exceed 1 cm. In fact, the majority are smaller than 5 mm. In most cases, the thickness does not exceed 1 mm, and as the width increases so does the thickness, the value of which never exceeds the width (Supplemenatry Table 5 and Supplementary Fig. 4). Because of the type and dimensions of the butts, the external and internal knapping angles could not be recorded.

When analysing the dorsal face, cortex is also absent and this side tends to follow convex delineations, although straight and uniangular are also relatively common. Quantifying the number of previous scars was also challenging, but on those where it was recorded, we found that the external sides usually do not carry more than 3 negatives. Finally, in terms of the morphologies, although the most represented frontal shape is trapezoidal, there is no predominant pattern since polygonal and semioval or oval are also frequently represented. Nonetheless, the small and microflakes clearly have triangular sagittal and transversal cross-sections (Supplementary Table 6).

All in all, the lithic products analyzed correspond to resharpening flakes. These were detached from the edge of a tool that has lost its sharpness and cutting efficency due to repeated use. With the reworking of the edge removing the blunted margin, the tool can continue to be used. The microdebitage removed carries the previous traces of utilization on the external platform edge and along the dorsal proximal retouch scars.

### Traceological analysis

We carried out a preliminary observation at low and high magnification of the 57 unretouched flakes for assessing their state of preservation. Overall, the specimens exhibited very pristine surfaces. Indeed, small and microdebitage flakes showed very fresh surfaces, except for a few flakes with light alterations in the form of a diffuse sheen, which did not hamper microscopic analysis. We recorded moderate and heavy patinated and rounded surfaces on two debris and two natural fragments. The latter are sometimes the result of site formation processes and the use of natural frost shards and frost debris as a raw material for tool production^22,23^. Furthermore, we recorded shiny scratches with random distribution on several microflakes due to contact with the metal sieve during their recovery. However, these marks did not create any analytical issues during the use-wear analysis.

#### Small and Microdebitage assemblage

**Figure 3:**
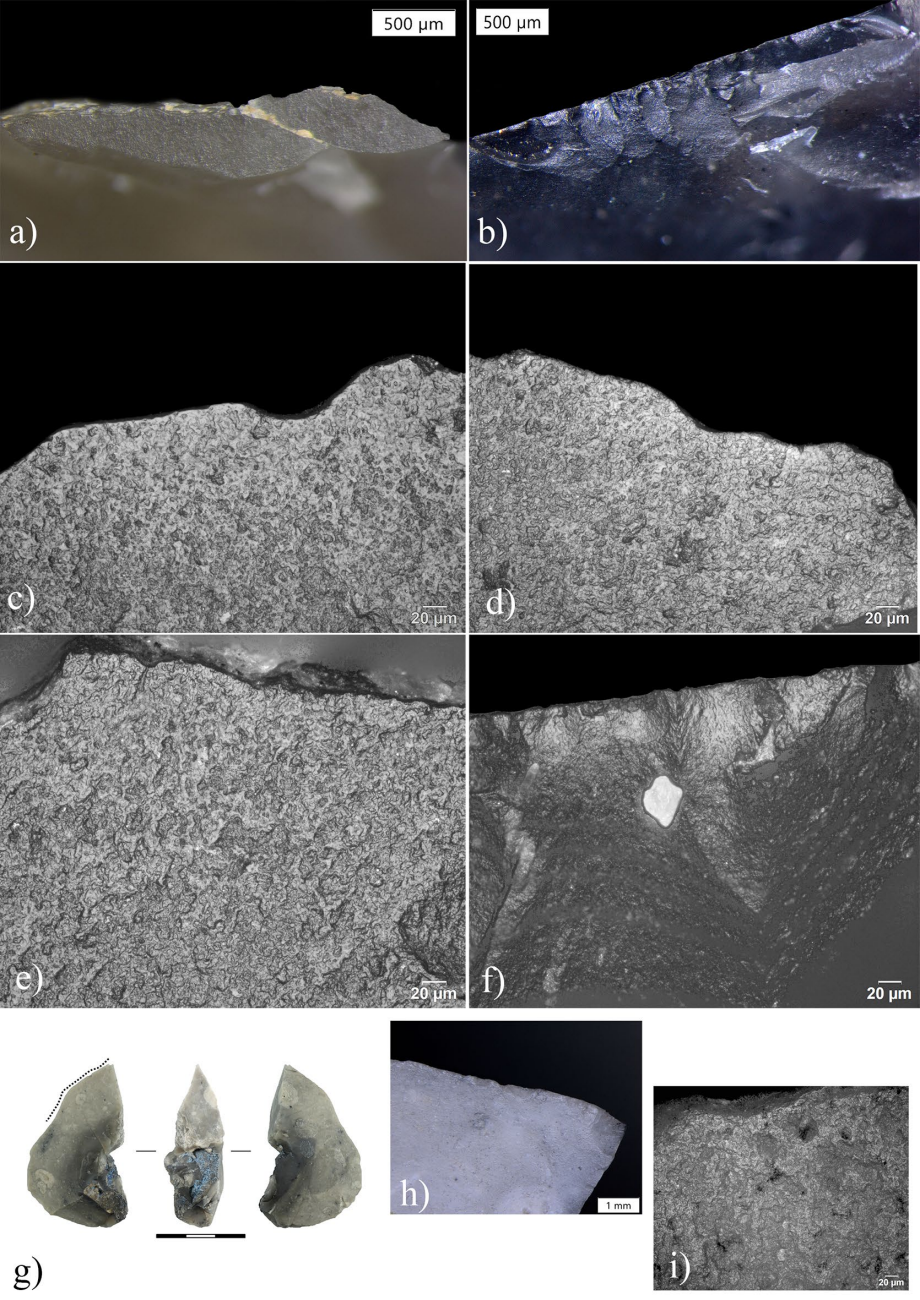
Archeological macro and microscopic use-wear traces on lithic artifacts from the elephant area at Schöningen 13 II-3. (**a**) view of the butt of the resharpening flake ID 28802 with a well-developed rounding along the external platform edge and yellow-orangish use-related microresidues (Magnification: 56x); (**b**) micro-edge damage developed along the dorsal face of the resharpening flake ID 29711 (Magnification: 56x); (**c–e**) micro-polish recorded on the butt of three resharpening flakes (ID 30876, 30878, 29716, Magnification: 500x) and interpreted as evidence of working fresh wood; (**f**) micro-polish recorded on the dorsal outer edge of the resharpening flake ID 29711 (Magnification: 500x); (**g**) natural flint fragment ID 29967; (**h**) edge damage along the natural sharp edge (Magnification: 25x); (**i**) micro-polish interpreted as related to the processing of fresh animal tissues, including hide (Magnification: 500x).

Out of the 57 small and microdebitage flakes, we found diagnostic microwear on 13. These microflakes show a combination of similar microwear attributes namely: (1) medium degree of edge-rounding, (2) a rough polish texture, (3) a granular polish topography with a wet appearance and a tight linkage and (4) a transverse and slightly oblique polish extended over the external platform edge (Fig. 3). No striations were found (Supplementary Table 7).

This polish combination was mostly recorded on the striking platforms, corresponding to the ventral face of the original tool (Fig. 3a). Rarely, polish developed on the dorsal outer edge inside the retouch scar (Fig. 3f and comparison on Supplementary Fig. 15g). Polishes extended from the external edge of the platform towards the center of the butt in a perpendicular and slightly oblique direction (Fig. 3c–e). The distribution appears continuous along the external platform edge if the resharpening flake was one of the first detached during resharpening. If the microflake instead corresponds to a “secondary” flake removed after a first one was knapped in the same location, we recorded spotted polishes at both ends of the external platform edge^18^ (see Supplementary Fig. 16 and Supplementary Information for more details).

Edge damage along the proximal dorsal edge of the resharpening flakes was rarely observed (Fig. 3b). When micro scarring is developed, we recognized both cone and bending initiation with feather and hinge terminations. Moderate edge rounding along the external platform edge of the flakes was recorded on 4 artifacts.

We did not record any reliable microwear (i.e., striations) that could match the clear technological traces produced by hammers in our experimental program and also reported in other experimental studies^24,25^. Nevertheless, we recorded a polish configuration on the striking platform of a flake (ID 30874) that might suggest a superimposition among technological and functional microwear (Supplementary Fig. 5c).

Overall, the set of observations discussed above match the attributes found on flakes used for woodworking, more precisely for processing fresh soft wood (e.g., spruce, pine, birch; see Supplementary Information for a comparison and Supplementary Figs. 15f,g and 17b). The degree of polish extension and the low rates of microwear recorded on the dorsal flake’s surfaces suggest the use of the original tools in a transversal motion.

#### The remaining part of the lithic assemblage

The rest of the sample subjected to analysis includes 4 debris pieces and 3 natural fragments. None of the debris exhibit microwear ascribable to production or to their subsequent use after detachment.

Among the three natural fragments, only one of them (ID 29967) exhibits macro and micro traces related to use (Fig. 3g). The piece (55 × 30 × 20 mm, 28.9 g), which has a sagittal and transversal cross-sections, shows a pointed morphology with a sharp and acute natural edge produced by a frost breakage. The acute edge is opposed to a natural back, which probably facilitated grasping by hominins.

We observed edge damage localized in the middle and distal part of the sharp edge associated with a medium degree of rounding (Fig. 3h). The tip also exhibits perpendicular damage that might have been produced during use. Under high magnification, we observed a distinct polish with a rough texture and a greasy appearance distributed along the active edge where edge damage is present. The texture and appearance of the polish suggest contact with fresh, fleshy animal tissues, including hide (Fig. 3i).

### Residue analysis

During *in-situ* optical observations, we recorded two types of residues: post-depositional accumulations and use-derived residues.

We identified use-derived residues on 13 micro and small flakes. On nine of them, residues appeared in combination with microwear, while four exhibited microresidues alone.

On all the artifacts, residues appeared optically homogenous in their spatial distribution and morphological attributes. All of them exhibit the same appearance, color, morphology and spatial pattern of distribution, although, the quantity of the deposition varied from piece to piece.

**Figure 4:**
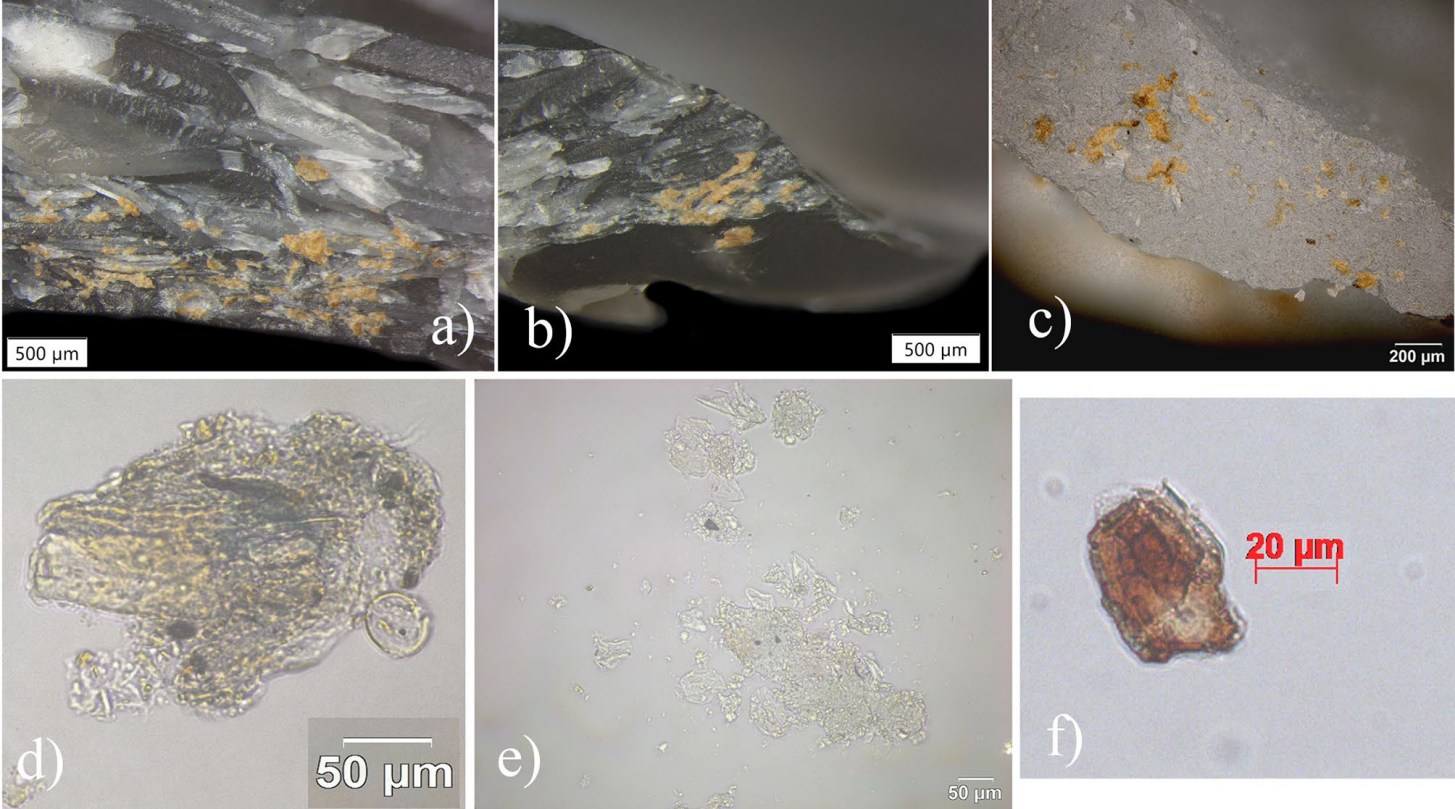
Archeological macro and microscopic residues identified on the lithic artifacts from the elephant area at Schöningen 13 II-3. (**a–c**) macroscopic use-related residues embedded and smeared in the retouch scars of the resharpening flakes ID 30251, ID 29716 (Magnification: 56x) and on the butt of resharpening flake ID 30255 (Magnification: 200x); (**d–f**) extracted micro-use related residues consisting of wood tissues and cortex particles observed in transmitted and polarized light on microflake ID 30251 (Magnification: 500x (**d,e**) and 400x (**f**).

**Figure 5:**
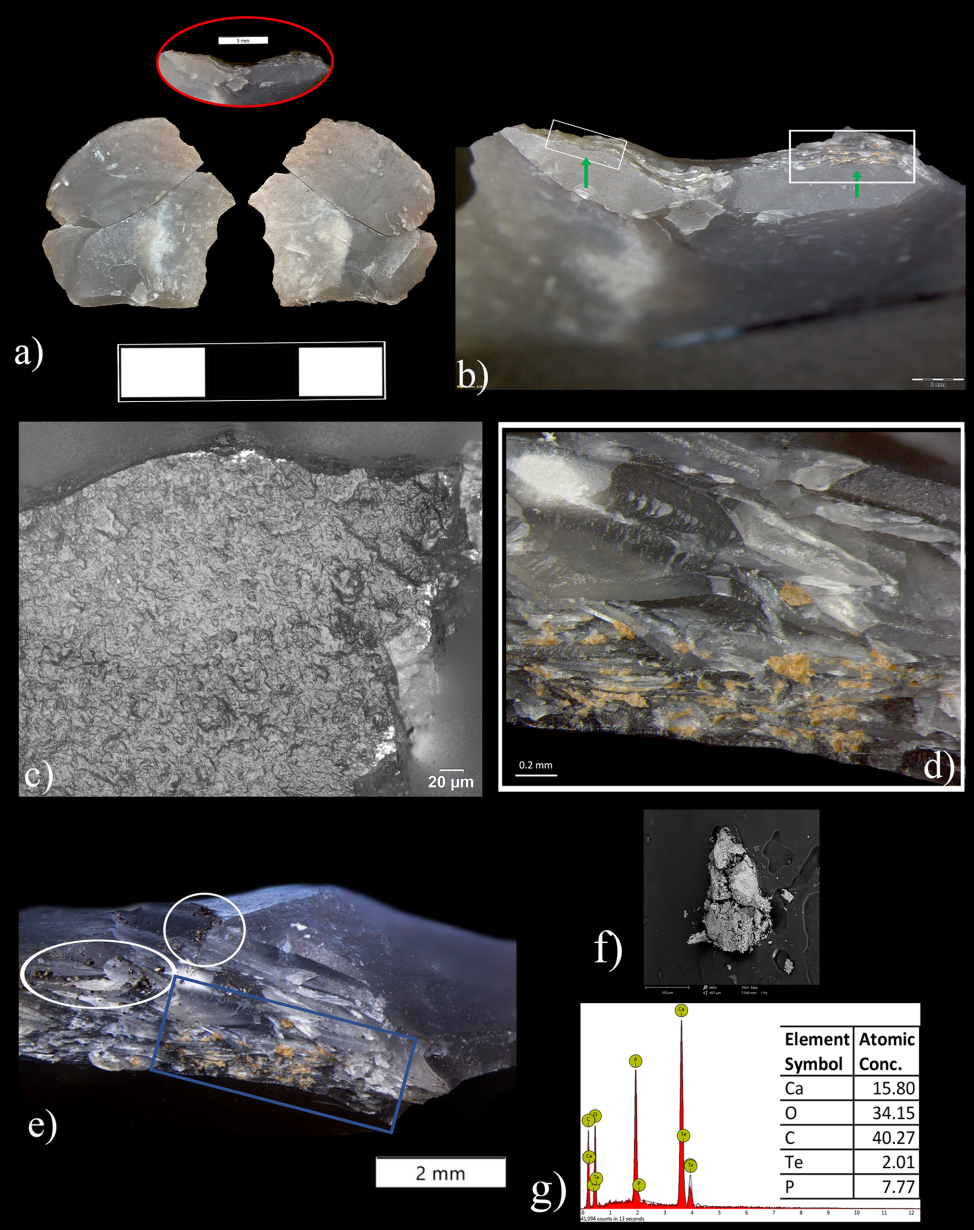
Archeological resharpening flake from the elephant area at the site Schöningen 13 II-3. (**a**) conjoin between ID 30251 and ID 29675; (**b**) butt of the small flake ID 30251 showing the external platform edge (green arrows) and the location of residues (white rectangles, Magnification 8x); (**c**) weak developped polish interpreted as not sufficiently diagnostic (Magnification: 500x); (**d**) micro use-related residues interpreted as tiny woody remains (Magnification: 500x); (**e**) dorsal retouch scars of small flake ID 30251 before cleaning (Magnification: 200x). Notice the clear difference between the use-related residues (blue square) and the micro blackish sediment particles (white circles), (**f,g**) extracted residue imaged using SEM and related elemental composition.

**Figure 6:**
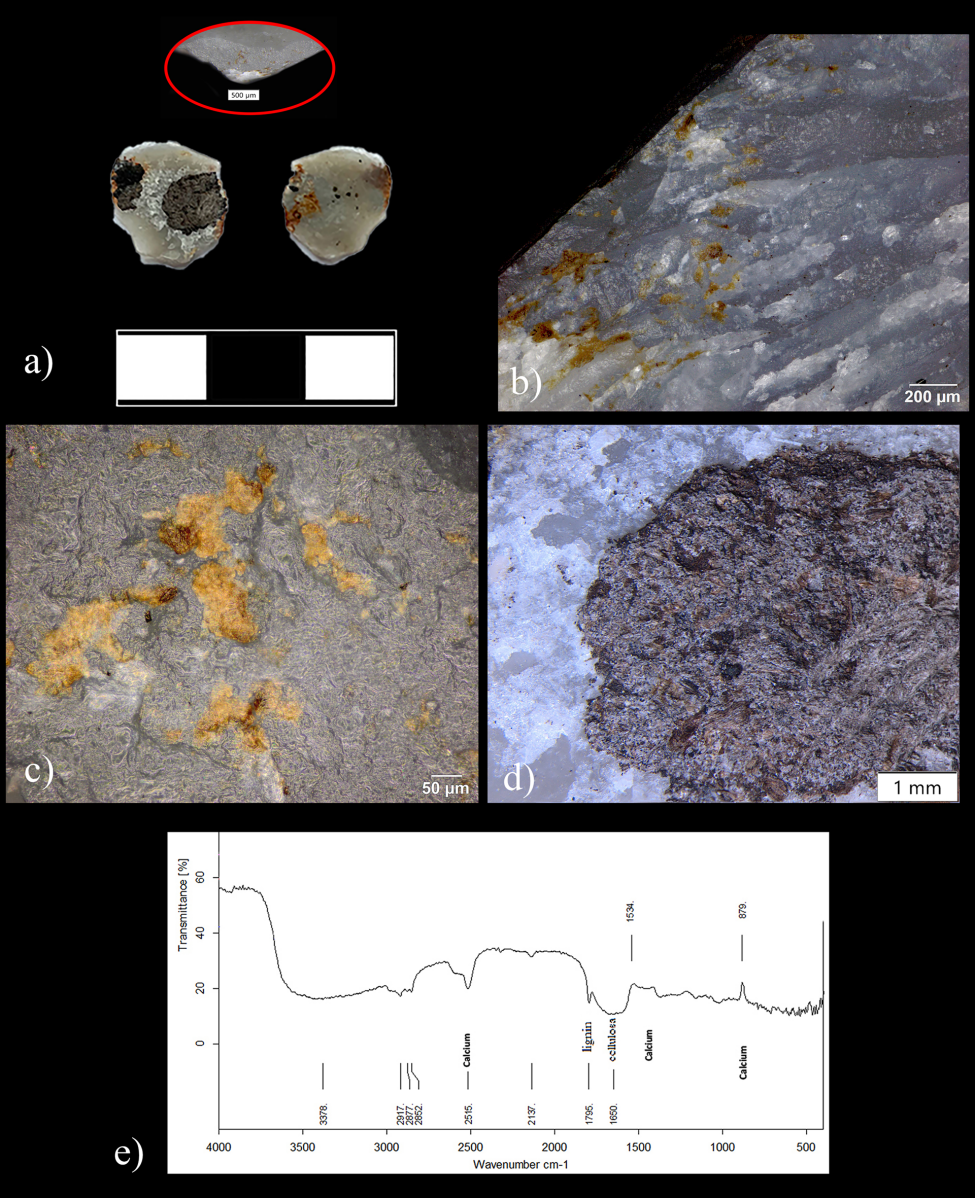
Archeological resharpening flake from the elephant area at the site Schöningen 13 II-3. (**a**) microflake ID 30255; (**b**) microresidues stuck on the dorsal proximal retouch scars and interpreted as tiny woody remains (Magnification: 200x); (**c**) microresidues of wood adhering on the butt (Magnification: 500x, dark field); (**d,e**) soil particles adhering to the dorsal surface and related FTIR spectroscopic absorptions showing a high concentration of calcium carbonate (Magnification in (**d**): 12.5x).

We recorded amorphous yellowish-orange residues with brownish shades firmly adhered on the striking platforms and/or sticking to the dorsal proximal retouch scars (Fig. 4). Three micro and small flakes (ID 30251, ID 29716 and ID 30255) show the densest cluster of residues (Fig. 4a–c, Figs. 5, 6 and Supplementary Fig. 6). On one (ID 29716), the microresidues are visibly smeared inside the retouch scars, a result of the kinematics and compression forces that occurred between the cutting edge of the original tool and the worked material processed during the activity (Fig. 4b and Supplementary Fig. 6c,e).

Interestingly, two resharpening microflakes (ID 30252; ID 28802) exhibited partially detached micro flint chips on their striking platforms and dorsal surfaces, originating from the kinematics of tool use during the activity. Both resharpening flakes showed the presence of use-derived residues trapped inside the fissure created at the interface between the resharpening flakes and the chip (Fig. 3a and Supplementary Fig. 7b). We recorded the same occurrence on experimental resharpening flakes used to process wood (Supplementary Fig. 7e).

Although residues show physical and spatial attributes compatible with use-wear, they lack diagnostic, morphological criteria when observed in reflected light. For a better understanding of their nature, we extracted the microresidues from a small flake (ID 30251) for observation in transmitted light. The results confirmed the use-wear interpretation. We identified plant tissues and microparticles of wood cortex, as confirmed by comparison with the reference collection and published literature (Fig. 4d–f, Supplementary Fig. 17c–e and comparison with^26,27^).

For further corroboration, we tested the same residues on the flake ID 30251 using two spectroscopic techniques: FTIR and SEM–EDX. The extremely small size particles of the residues inhibited reliable measurements with *in-situ* FTIR analysis. However, the results from the X-ray analysis match the elemental composition of modern wood tissues (i.e., spruce), showing high concentrations of carbon (C) and oxygen (O), the two dominant elements in organic molecules (Fig. 5f,g and comparison in Supplementary Fig. 17f,g). Nevertheless, a high peak of calcium was also recorded. Although a minimum percentage in weight of calcium is typical in wood and vegetal tissues (Supplementary Fig. 17f,g), the large amount of deposited calcium carbonate has an exogenous origin and correlates with the taphonomy of the site. Underground water rich in calcium carbonate caused the deposition of a thin layer of calcium observed on both finds and sediment particles. The deposition of calcium carbonate on top of the residues proves their antiquity and excludes the possibility of modern contamination. Calcium depositions have also been observed dispersed across the surfaces of a few specimens (Fig. 6a,d and Supplementary Fig. 6a).

High absorption of calcium was also confirmed by the FTIR measurement performed on a patch of soil covering one microflake tested as a control sample (ID 30255) (Fig. 6e). A thin granular whitish layer of calcite covering the soil particles was also observed at low magnification (Fig. 6d).

Additionally, the FTIR results showed the plant-rich nature of the sediment itself (also observed with optical microscopes). Despite that, micro-wood residues identified on the resharpening flakes exhibit morphological features, a pattern of spatial distribution alongside a strict correlation with the use-wear traces that securely attest to their use-derived origin, eliminating the possibility of incidental deposition.

All in all, the nature of the organic residues supplements and reinforces the functional interpretations garnerd from use-wear analysis (Figs. 5, 6 and Supplementary Figs. 6–9). The firm correlation between traces and use-derived residues observed on nine micro and small flakes let us confidently interpret the other 5 microflakes exhibiting microresidues, but weak and not sufficiently diagnostic polishes, as part of the same techno-functional cycle.

The combination of use-wear and residue analysis also supports previous results by Rots and colleagues^28^ who identified the processing of animal materials and wood as the main tasks performed at Schöningen.

## Discussion

The archaeological complex of Schöningen stands out as one of the most significant Middle Pleistocene sites in Europe. Thanks to the exceptional preservation, the rapid burial of the finds and the spatial distribution of the material, the ca. 64 m^2^ studied area at Schö 13 II-3 offers a rare opportunity to investigate a snapshot of hominin activities and subsistence behavior during MIS 9 in Northern Germany. The results presented in this study, along with very pristine archeological deposits, have allowed us to reconstruct what might have happened around the elephant spot ca. 300,000 years ago.

The border of the lake of Schöningen was repeatedly visited by hominins over millennia. This is proven by the numerous archaeological sites, some of which are the result of repeated visits^29^. During one of these incursions, the hominins found the carcass of a ca. 50 year old, probably female elephant who likely died of natural causes^30^. There, they used at least one sharp-edged natural flint fragment to butcher the carcass, possibly transporting the left front and the distal hind legs to a secondary location. It is well-known that the head and feet contain high nutritional values but, given their complex structure, require high time and energy investments to be processed^31–33^. For this reason, hominins may have transported these portions to a camp-site for secondary processing^34^. So far, preliminary observations of the Schöningen elephant have no revealed cutmarks, but further restoration and study are underway (Ivo Verheijen, pers. Comm.).

Given the great amount of meat and fat provided by an elephant, it is safe to suggest that additional, perhaps larger, more sophisticated tools were used during butchery. So far, no tools matching the studied microdebitage have been discovered. This suggests that selected tools were kept and transported to other locations for resharpening and reuse, instead of being abandoned. This implies the hominins’ desire to prolong the use-life of selected tools by adopting maintenance procedures and curation. This assumption is confirmed by the results provided in this study.

Through a comprehensive morphotechnical and functional analysis we establish that the small and microflakes found among the elephant’s bones are resharpening flakes originating from tools previously used to process fresh wood. The anatomy of the resharpening flakes (e.g., ventral and dorsal faces, platforms, bulbs, axis lengths, flint color, flint inclusions) suggests that at least two tools were resharpened on the spot of the elephant after previous use.

The lack of clear hammer percussion marks on the striking platforms might reflect differences in flintknapping techniques and gestures used by the Schöningen hominins. This hypothesis deserves more comprehensive experimental studies in the future. However, the recovery of three bone retouchers with embedded flint chips^30,35^ (Ivo Verheijen, pers. Comm. and see also experimental results in the Supplementary Information) firmly suggests the use of soft hammering techniques. This is also inferred by the results of the morphotechnical analysis on very small flakes, which are quite thin and flat, and tend to carry lips or feathered termination.

The clear match between the use-wear traces and the use-derived microresidues documents the production and/or maintenance of wooden tools and perfectly fits the wood technology widely demonstrated at Schöningen 13 II-4, at the well-known “Spear Horizon”^36,37^. It is difficult to establish whether woodworking activities at Schö 13 II-3 were contemporaneous with the butchery of the elephant. It is also possible that the stone tools were used to process wood at another time or location before being brought to the elephant and resharpened for a new purpose. Certainly, the Schöningen hominins during this visit preferred to prolong the use-life of formal tools for future tasks instead of abandoning them after use. In parallel, they also chose to use simple naturally sharp fragments to satisfy more immediate needs in an expedient way. Although flint quality was good, it suffered incipient frost fractures that made flaking unpredictable. This aspect may explain the need to resharpen tools and the abundance of microdebitage observed at Schöningen 13 II-3 and 13 II-4.

The implementation of expedient and curational behaviors in the production and use of the tool-kits testifies to the versatility, flexibility and plasticity expressed by the Schöningen hominins while interacting with their environment. This is reflected not only in the production and use of the lithic material but also in the exploitation of plants and animals. The production of sophisticated wooden hunting weapons and the short use-life of the bone retouchers illustrate the duality of this techno-functional behavior^29,38^. The way in which different raw materials were conceived and integrated by the Schöningen hominins within their technological repertoire follows a mental template, which resulted from their cultural heritage acquired and transmitted across generations. This cultural inheritance is the result of the continuous interactions between culture, biology and ecology in the form of a triple inheritance that has shaped human beings since the dawn of human evolution^39,40^.

## Conclusion

This work is the first comprehensive and integrated techno-functional study conducted on Lower Paleolithic microdebitage. Through a careful and in-depth analysis of tiny resharpening flakes we provide a high-resolution glimpse into the life of 300,000-year-old hominins on the shore of the paleolake at Schöningen. Microartifacts have revealed unexpected results providing insight into tool production, maintenance and function, diversification of activities and exploitation of resources. The area of study within the locality Schö 13 II-3 proved to be a key spot for investigating how hominins interacted with the interglacial lakeshore environment and how they implemented organic and inorganic resources into their technological repertoire. In this respect, the results from the analysis of the microdebitage brought to light direct and indirect evidence for the interrelated and mutual use of lithic, osseous and plant technology.

In addition, the pristinely preserved lithic assemblage in direct association with an almost complete skeleton of a straight-tusked elephant at Schö 13 II-3 provides a unique opportunity to investigate the hominin-faunal interactions and, in particular, the relationships between early hominins and megafauna. Despite the documented importance of proboscideans in hominin subsistence strategies, little is known about the way these large pachyderms were hunted and exploited by early hominins (for an overview see^41–43^ and references therein). Our results, combined with the ongoing faunal analysis of the elephant bones, will further illuminate this important question.

Alongside the significant contributions to our understanding of hominin subsistence activities and techno-functional behaviors during the Middle Pleistocene, this study also reassesses and highlights the importance of studying microdebitage. We demonstrate that microartifacts do not contain less information than their larger counterparts. To the contrary, they constitute a key resource for understanding early hominin lifeways. This is especially relevant in contexts where no tools are recovered or at sites characterized by low numbers of lithic tools, such as Schöningen. In this regard, our example opens new perspectives for reconstructing hominin behavior based significantly on the recovery of small and microartifacts.

## Materials and methods

The material presented in this study consists of 57 small and microdebitage flakes, 4 debris pieces and 3 natural fragments. Many of these artifacts were found among the elephant bones, while others were lying in the immediate vicinity of the skeleton, in an area currently corresponding to ca. 64 m^2^.

Only 37.5% of the lithic assemblage was found in situ, the other 62.5%, all corresponding to microdebitage, was found during the sieving process after water screening of the collected sediments. After collection during excavation or in the lab, each piece was packed in a plastic bag with a label and stored in boxes until analysis. For that, the lithic remains were studied macroscopically, using different hand lenses and under low-power magnification with the stereomicroscope *MOTIC SMZ 171* (with 10 × ocular magnification and 0.75 × −5 × zoom range). Artifacts were always handled with powder-free nitrile gloves.

The lithic assemblage was analysed following the Logical Analytical System^44,45^, a methodology that focuses on the technical characteristics generated during the production process, which are seen in the final morphology of the artifacts. Technological and morphological measurements were also recorded.

### Use-wear analysis

We carried out the traceological analysis at the Material Culture Laboratory (hereafter MCL) at the University of Tübingen. We combined low and high power approaches, following well-established methodology used in functional studies^46–53^. For observation at low magnification, we used an Olympus SZX7 stereomicroscope with magnification ranging from 8 × to 56 × and equipped with a LED ring light source. Based on the characteristics and distribution patterns of edge damage and rounding, we defined the use motions and the hardness of the worked materials^46–48^.

We performed more exact identification of the activities and processed materials by recording polish, striations and microrounding at magnifications of up to 500x. We recorded and described the texture, topography, distribution and linkage of microwears, following the common qualitative criteria used for the high power approach^49–53^. For this purpose, we used an Olympus BX53M metallographic microscope with vertical incident light.

The majority of the archeological specimens were recovered after water screening, but the artifacts were not manipulated underwater with the purpose of cleaning their surfaces prior to this study. In addition, they were always handled with powder-free nitrile gloves during the entire analytical procedure.

We based the use-wear interpretations on the results of *ad-hoc* experimentation (see Supplementary Information), specifically designed for this case study. Additional comparisons were performed based on the experimental collection available at the MCL and the available literature coherent with this case study^18,20,54,55^.

### Residue analysis

We observed and recorded the residues on the archeological material before and after cleaning. The double observation was important, as it allowed us to confidentially identify the reliable, strongly adhering residues. The residues that disappeared after cleaning were considered non-use related.

We recorded and characterized microresidues related to use as well as residues accumulated by post-depositional agents.

To identify both classes of residues, we used different techniques (e.g., *in-situ* observations, pipette extraction, scalpel extraction) and other combined approaches (optical observations, elemental and spectroscopic characterization). Residues were characterized paying particular attention to their morphological attributes (appearance, color, consistency, inclusions), their distribution, location, directionality and link with use-wear traces.

We first characterized the archeological residues *in-situ* with the aforementioned Olympus reflected optical light microscopes (RL) using various lighting conditions (i.e., brightfield, darkfield, DIC/polarized light).

We thus selected one specimen with the most abundant residue accumulations for a subsequent observation in extracted solutions. For this purpose, we placed 20 μL of distilled water in the area of residues (˜7 mm) and we extracted the material with an adjustable pipette. We mounted extractions onto clean glass slides with a 50% solution of distilled water and glycerol. We documented residues in transmitted light (TL) using a Zeiss Axio Imager Petrographic Microscope equipped with plane-polarized and cross-polarized light at the Laboratory available at the Department of Geoarchaeology at the University of Tübingen, and an Olympus BX53M metallographic microscope in transmitted light along with the 3D digital microscope Hirox HRX-01 covering a magnification range of 20 × -2500 × available at the MCL at the University of Tübingen.

We later subjected the same specimen to spectroscopic characterization of residues using X-ray (SEM–EDX) and Fourier Transform Infrared spectroscopy (microFTIR).

For SEM–EDX analysis, we carefully and lightly scraped a few microparticles of residue with a scalpel from the specimen and placed them onto a standard SEM carbon adhesive glued on an aluminum stab. The extracted residues were observed without coating in a high-vacuum mode and working distance between 122 and 923 μm using a Phenom XL Scanning Electron Microscope from Phenom-World, Eindhoven—Netherlands equipped with cerium hexaboride (CeB6) source, and Phenom —element Identification Software at the Senckenberg Center for Human Evolution and Palaeoenvironment at the Department of Geosciences at the University of Tübingen.

We used different accelerating voltages during the analysis of residues: 5 kV was used to characterize topographic and textural traits, while 15 kV mode provided elemental information through grey-scale images according to the atomic number using the high sensitivity backscattered electron detector.

For microFTIR we used a Bruker Optic Alpha-R portable interferometer with an external reflectance head covering a circular area of about 5 mm in diameter at the Laboratory of Technological and Functional Analyses of Prehistoric Artifacts at Sapienza University of Rome (LTFAPA). The investigated spectral range was equal to 7000–375 cm^−1^, with a resolution of 4 cm^−1^, and at least 250 scans were performed.

Measurements were performed in situ. We placed the samples directly in front of the objective, without preliminary treatments, and took infra-red measures on at least three points for each analysed residue.

The interpretation of residues was supported by the results of the experimental program (see Supplementary Information and Supplementary Figs. 10–14 and 17) and by a large FTIR and EDX spectra reference collection of experimental microresidues on stone tools. Additional comparisons were also made with available published and unpublished literature^26,56–63^.

### Cleaning protocols

#### Archeological materials

We gently and shortly immersed the individual archeological artifacts in demineralized water and 2% neutral phosphate-free detergent (Derquim®) in a Petri dish to contain them during cleaning. Subsequently, they were rinsed in fresh demineralized water^64^.

The flakes exhibiting micro-residues were only rinsed in fresh demineralized water. Occasionally, we used ethanol/acetone for cleaning tiny portions of the flake’s platforms during the use-wear analysis.

#### Experimental materials

After recording the microresidues, we cleaned the experimental microdebitage with a dilute 3% acetic acid solution (CH_3_COOH) for 10 min, and then in a dilute 3% sodium hydroxide (NaOH) base for 10 min. We then rinsed the flakes with demineralized water in an ultrasonic tank for 5 min. These procedures allowed us to remove all organic matter from a microartifact’s surface and prepare the sample for the subsequent use-wear analysis.

## Supplementary Information

Supplementary Information.

## Data Availability

All data generated or analysed during this study are included in this published article [and its Supplementary Information files].
