# Supplementary material for: Using microartifacts to infer Middle Pleistocene lifeways at Schöningen, Germany
Source: Sci Rep. 2022 Dec 15;12:21148. doi: 10.1038/s41598-022-24769-3 (PMC9755147; doi:10.1038/s41598-022-24769-3)
Supplement: Supplementary file 1 — Supplementary Information. [file 41598_2022_24769_MOESM1_ESM.pdf]

Using microartifacts to infer Middle Pleistocene lifeways at Schöningen, Germany

Flavia Venditti[1], Bárbara Rodríguez-Álvarez[2], Jordi Serangeli[2], Stella Nunziante Cesaro[3], Rudolf Walter[1], Nicholas J. Conard[1,2,4]

[1] *Department of Early Prehistory and Quaternary Ecology, University of Tübingen, Schloß Hohentübingen, Burgsteige 11, 72070, Tübingen, Germany.*
[2]*University of Tübingen, Senckenberg Centre for Human Evolution and Palaeoecology, Paläon 1, 38364, Schöningen, Germany.*
[3] *Scientific Methodologies Applied to Cultural Heritage (SMATCH), Rome, Italy.*
[4]*Senckenberg Centre for Human Evolution and Palaeoecology, University of Tübingen, Rümelinstrasse 23, 72070, Tübingen, Germany.*

Corresponding author: flavia.venditti@uni-tuebingen.de

Supplementary Information

Microartifacts

Microartifact is a general term used to define tiny remains of material culture that enter the archeological record following hominin activity[3,4,9,65]. Microartifacts may result from different processes such as accidental breakage, trampling, tool use, tool production and maintenance. Any of these processes provide a different set of information.

For many years stone artifacts smaller than 2 cm were overlooked when classifying and studying lithic assemblages since they were considered debris or waste unable to provide any relevant information[66]. However, in the last decades, more attention has been paid to the small fraction of lithic industries at different archaeological sites[31, 67, 68, 69], and techno-functional studies have shown that small and microflakes could be the desired product of lithic production and carry traces of use[15,18,70,71,72,73,74,75].

In this study, we focus on microartifacts removed from the use-edge of tools after resharpening to maintaining a cutting edge (hereafter resharpening flakes), which are defined as microdebitage. We refer to chips as flakes smaller than 5 mm, microflakes ranging from 5 mm and <15 mm, and small flakes between 15 and <30 mm.

Schöningen 13 II-3 and the area of the elephant

The Middle Pleistocene archeological site complex of Schöningen comprises of over 40 lacustrine sedimentary layers with a total depth of 7.5 meters[76]. The greater sedimentary cycles have been interpreted as silting sequences reflecting changes in the level of a paleo-lake[77]. The archaeological complex includes several Lower Paleolithic open-air sites dated to the Reinsdorf Interglacial (MIS 9, ca. 300,000 years) characterized by outstanding preservation[29].

One site, where we are currently working, is Schöningen 13 II-3. This site covers a wide extension of so far ca. 1000 m² (Supplementary Fig. 1a). Since the beginning of the excavations in 1994, we recovered several find concentrations and numerous remains of large mammals as well as lithic artifacts and a potential wooden artifact[78].

Between 2017-2020, in an area corresponding to ca. 64 m^2, we discovered an almost complete skeleton of an extinct Eurasian straight-tusked elephant in the silting event 3, layers 3b, 3bc and the upper surface of Schöningen 13 II-2a (preliminary study[30], Supplementary Fig. 1, middle and right pictures). In that area, we recovered several dozen small and microartifacts, most of them between 5 and <15 mm, along with 4 pieces of angular debris and 3 natural fragments. Although it may seem that the sample is scarce, we would like to point out that, over the years, we have always taken the exact same care in excavating, water screening and analysing the sediments, and no lithics have been found in Schöningen 13 II-3. Hominin activity is also testified on the spot by three bone retouchers used for flint knapping, two of which refit together but were used as two separate tools (Ivo Verheijen, pers. comm.). Many of these artifacts were found among the elephant's bones, while others were lying in the immediate vicinity of the skeleton. The elephant has not been comprehensively studied yet, there are numerous bones undergoing restoration and zooarcheological analyses are still in process. A specific article on this elephant is in preparation.

The area with the elephant represents a single, limited event in time and space. This peculiarity along with the pristine conditions of the recovered finds makes Schöningen 13 II-3 a key site for providing a high-resolution picture of hominin behavior during the Middle Pleistocene.

The experimental program

The archeological interpretations were supported by the results of a specific experimental program designed at studying the morphological attributes and distribution patterns of techno-functional microwear and microresidues on the resharpening flakes.

Experimental design

The locality of Schöningen is a complex of sites with a low density of lithic artifacts, in many cases, appearing as isolated finds, with the exception of a richer accumulation at the Spear Horizon[22]. At the elephant area in Schö 13 II-3, we did not recover any tools matching the microdebitage found around the carcass. However, since scrapers are common across the broader site complex, especially at Schö 13 II-4, we decided to produce and use scraper replicas to analyze the microdebitage produced by their resharpening. This choice was also dictated by the preliminary traceological results on the microdebitage, suggesting the performing of scraping activities.

One of the authors (RW) produced the experimental scrapers using high-quality, fine-grained Baltic flint collected on the coast of northern Germany. After use, the scrapers were resharpened by the same flintknapper.

The experiments were designed to process materials and perform activities that were most likely a part of the daily life of the Schöningen hominins. The scrapers were manually used by FV for a minimum of 60 minutes for scraping tasks, except for one tool used by combining transversal and longitudinal motions for 30 minutes (Supplementary Fig. 10).

In our experimental protocol, we included different animal and vegetal materials in both dry and fresh conditions (Supplementary Table 8 and Supplementary Fig. 10). As a reference, we also analyzed microwears on two flint replicas belonging to two different experimental programs and used to process wood (one scraper and one unretouched flake). Moreover, to create a control sample, we also examined microdebitage flakes from one unused scraper.

All of the experiments were performed under controlled conditions and all data were recorded. Once we concluded the practical activities, we observed the distribution of use-related residues on the scraper's active edges prior to resharpening at low magnifications.

Resharpening process and resharpening flakes

The flintknapper RW resharpened the used scrapers by recreating the original scalar retouch identified on some archeological specimens. We did not impose any specific resharpening technique or gestures and we gave the knapper the freedom to follow their feeling according to the morphology of the active edge and the type of hammer used.

We designed the experiments with the aim of testing soft and hard hammer percussion (Supplementary Fig. 10). This also allowed us to record technological microtraces and residues produced by the different hammers.

We used a dry horse metapodial as a soft, organic hammer (area on the epiphysis, Supplementary Fig. 10i) and a boxwood billet (Supplementary Fig. 10f) while two limestone hammerstones were used for testing hard hammer percussion (Supplementary Fig. 10j). The recovery of three bone retouchers in the elephant area with embedded stone chips proved that soft hammering techniques were in use at Schö 13 II-3[30]. Embedded flint chips stuck in the bony tissues after repetitive blows were also recorded in our experimental trial, as shown in Supplementary Fig. 10h.

We used only one type of hammer per each tool, except for one scraper that was resharpened using both wood and stone (see Supplementary Table 8). The choice of the hammer depended on the material worked by the tool. This means that we always used a hammer made of a different material than the one the scraper was used for. This has allowed us to easily recognize situations where technological residues overlapped with use-related ones[25].

We produced a total of 152 resharpening flakes on a total of 7 tools. The scrapers were considered fully resharpened when the whole dorsal active edge appeared fresh and without evidence of remaining used edge portion with visible use-traces or residues. For each scraper, we counted the total number of strokes necessary for complete resharpening, and we individually numbered and collected each resharpening flake after each strike. In several cases, more than one micro flake (up to 5) was removed with a unique stroke. We collected microflakes ≥ 2 mm in width to facilitate microwear analysis. Each flake was then placed in a clean plastic bag, awaiting microscopic analysis. The use-wear analysis was conducted using the optical equipment described in the Materials and Methods section in the main manuscript and available at the MCL at the University of Tübingen.

Results

In the next paragraphs we do not discuss the qualitative features of microwears and microresidues produced by working different materials with stone tools, these aspects being already extensively covered by several articles in the traceological literature[27,49,51,56,57,58,62,79,80]. Instead, we thought it would be more useful to discuss the location and distribution patterns of residues and wear traces on the microdebitage, being the two most relevant variables in the microscopic analysis of the resharpening flakes.

Residues: spatial patterns of distribution

After resharpening, we first established the spatial distribution of microresidues on the resharpening flakes. For this purpose, we oriented the flakes according to the technological axis with their dorsal sides facing up, and we divided the dorsal face (the only face we recorded traces and residues) into three segments following the methodological protocol of Lombard[81]: 1) proximal, 2) mesial and 3) distal (Supplementary Fig. 11). We thus recorded all microresidues and counted their occurrence in the above-mentioned segments. Clusters with large accumulations or very few fragmented residues were counted as a single occurrence (a note for a few accumulations was reported). In addition, we listed and recorded the platform as another potential area for residue accumulation.

Although we are conscious that this method is not quantitatively reliable, our aim here is to monitor the incidence and distribution of technological and used-related microresidues regarding 1) the material worked/used and 2) the artifacts' surfaces.

As a general impression, we noticed that organic residues accumulated more on the proximal segment of the resharpening flakes. Semi-dry hide and dry wood recorded the highest number of resharpening flakes with residues accumulated in this region, as shown in Supplementary Figs. 12 and 13. This is not surprising since it is the area in direct contact with the processed material. However, we recorded differences according to the material processed and its state of freshness.

Within vegetal materials, fresh and dry wood seem to be inversely proportional: we recorded more residues on the mesial and distal segments of resharpening flakes in contact with fresh wood, while dry wood residues accumulated more on the proximal area.

For the animal materials, the two scrapers used to process fresh skins also showed a reverse trend. Microflakes in contact with roe deer skin accumulated residues more in the distal area while those in contact with wild boar skin adhered to the proximal part. This may be due to the fact that the roe deer skin was wetter when processed, allowing residues to more easily slip away from the active edge of the tool during processing. Indeed, residues recorded on the microdebitage produced by the scraper used to process semi-dry hide accumulated more on the proximal area of flakes instead of on the mesial and distal ones.

The distribution patterns of bone residues are instead distinctive with the occurrence of bony tissues mixed with collagen present only on the proximal segment. Finally, fresh animal tissues after butchery are more represented in the proximal area of the resharpening flakes.

Microresidues also accumulated on the platform of several microflakes, with the highest number recorded for fresh skin and the lowest recorded for semi-dry hide.

We recorded not only use-related residues but also technological residues produced by retouching the tools with different hammers, which were found mostly superimposed on the use-related residues (Supplementary Fig. 14b,d,f). Occasionally, we found them disconnected as clearly distinguishable lines of deposition along the outer proximal edge (Supplementary Fig. 14d). Technological residues also accumulated on the platform, mostly along the external platform edge. They often showed a random distribution, even though we observed hammer percussion residues in bands with a clear directionality, indicating the impact direction during resharpening (for an overview on this topic see[25].

Microwear: spatial patterns of distribution

Microwear analysis of microdebitage is challenging. As reported by Chan and colleagues[18], the interpretation and identification of micro traces on resharpening flakes are more difficult than performing the same analysis on a complete active tool edge.

This is due to a series of factors including 1) the small size of the flakes and difficult handling under the lens of microscopes, 2) the limited area of traces that could be analyzed, 3) the superimposition of technological and functional traces on a limited analyzable area and 4) the dissociation of the resharpening flakes from the original tool's active-edge from which they have been removed. These aspects highlighted by Chan and colleagues[18] were also experienced during our microscopic analysis.

The above-mentioned factors have important implications on the distribution patterns of micro-wear on the resharpening flakes. Indeed, we noticed that the location of microwear is, in general, very specific and, in our replica, mostly restricted to the flake's platform (Supplementary Fig. 15a-f). This aspect certainly reflects the activity of scraping that we performed with the tools, with the ventral face (the striking platform of the resharpening flakes) being most in contact with the worked material. However, we also recorded microwear along the dorsal ridges; although, the texture and the topographical traits of polish were less informative in comparison to those developed on the platforms (Supplementary Fig. 15g). Traces on the dorsal ridges are particularly important when the butt of the resharpening flake is not preserved.

Concerning the microwear distribution, we observed that polish develops continuously along the external platform edge of the microflakes (in the luckiest cases), but they may also display a discontinuous arrangement or be not present at all. This was particularly observed when the micro flake under study was not the first one that was removed from the edge of the tool, but rather a "secondary" flake detached on the same spot where the previous one was knapped (Supplementary Fig. 16). This can arrive frequently during resharpening, depending on the degree and pattern of the resharpening-retouch. In the case of secondary flakes, the original use edge portion of the tool is retained only on the two lateral extremities of the external platform edge of the micro flake, as shown in Supplementary Fig. 16. However, their interpretation is not always so straightforward, especially when archeological materials are under study. We cannot forget that what was once the ventral face of the tool is now the striking platform of the resharpening flakes. This implies that, along with the use-wear signs, a flake's platform may also retain evidence of the manufacturing traces produced by the contact of the hammer with the stone tool during resharpening. Manufacturing traces are in general quite distinct with a clear directionality of polishes and can be easily distinguished from use-wear traces (Supplementary Fig. 14a,c, e and[24,51,80]). Nevertheless, when they overlap with functional traces on a very small portion of the lithic surface (such as on the striking platform) they may hamper the interpretation of functional traces. This is especially true when observing the archeological material because of the presence of other factors that may complicate the microscopic observations (e.g., post-depositional traces).

Supplementary Figures:

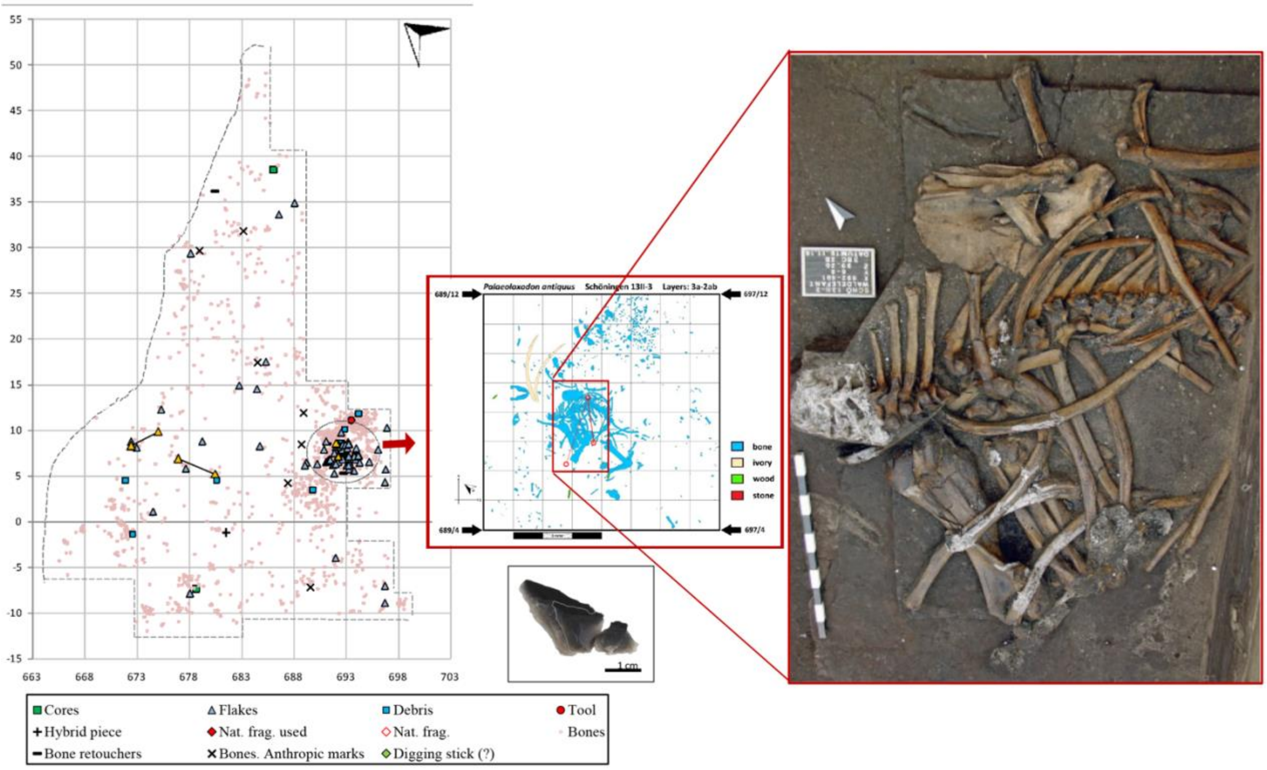

Supplementary Fig. 1. Distribution of the remains recovered so far from the whole excavated area from Schö 13 II-3 (left picture). Detailed drawing of the area where the elephant and most of the finds were found (picture in the middle with the two (+1) refitting pieces). Picture of the elephant bones as they were being excavated (right picture). Drawing: D. Mennella; Photo: J. Lehmann.

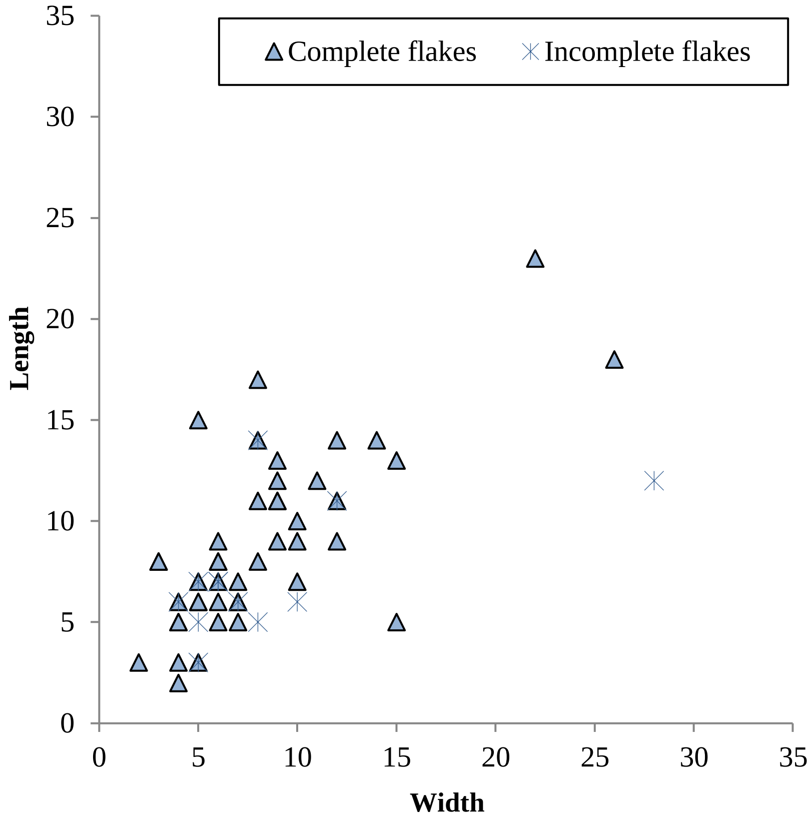

Supplementary Fig. 2. Dimensions of the unretouched flakes from the elephant area at Schö 13 II-3. Data in mm.

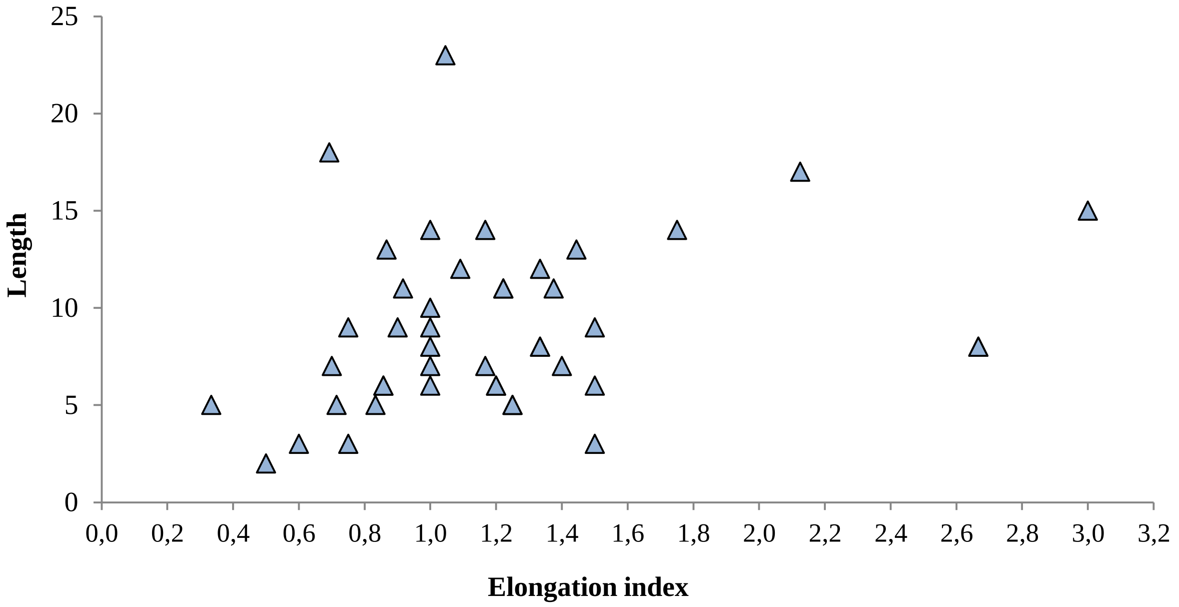

Supplementary Fig. 3. Relation between length and elongation index (L/W) of the complete flakes from the elephant area at Schö 13 II-3.

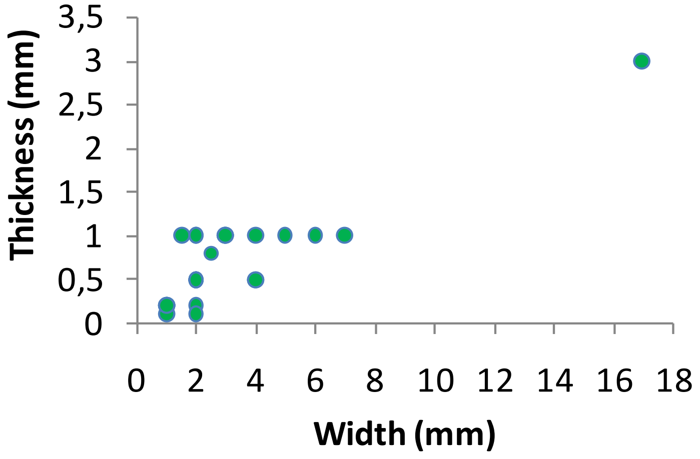

Supplementary Fig. 4. Width and thickness of the butts from the flakes around the elephant at Schö 13 II-3.

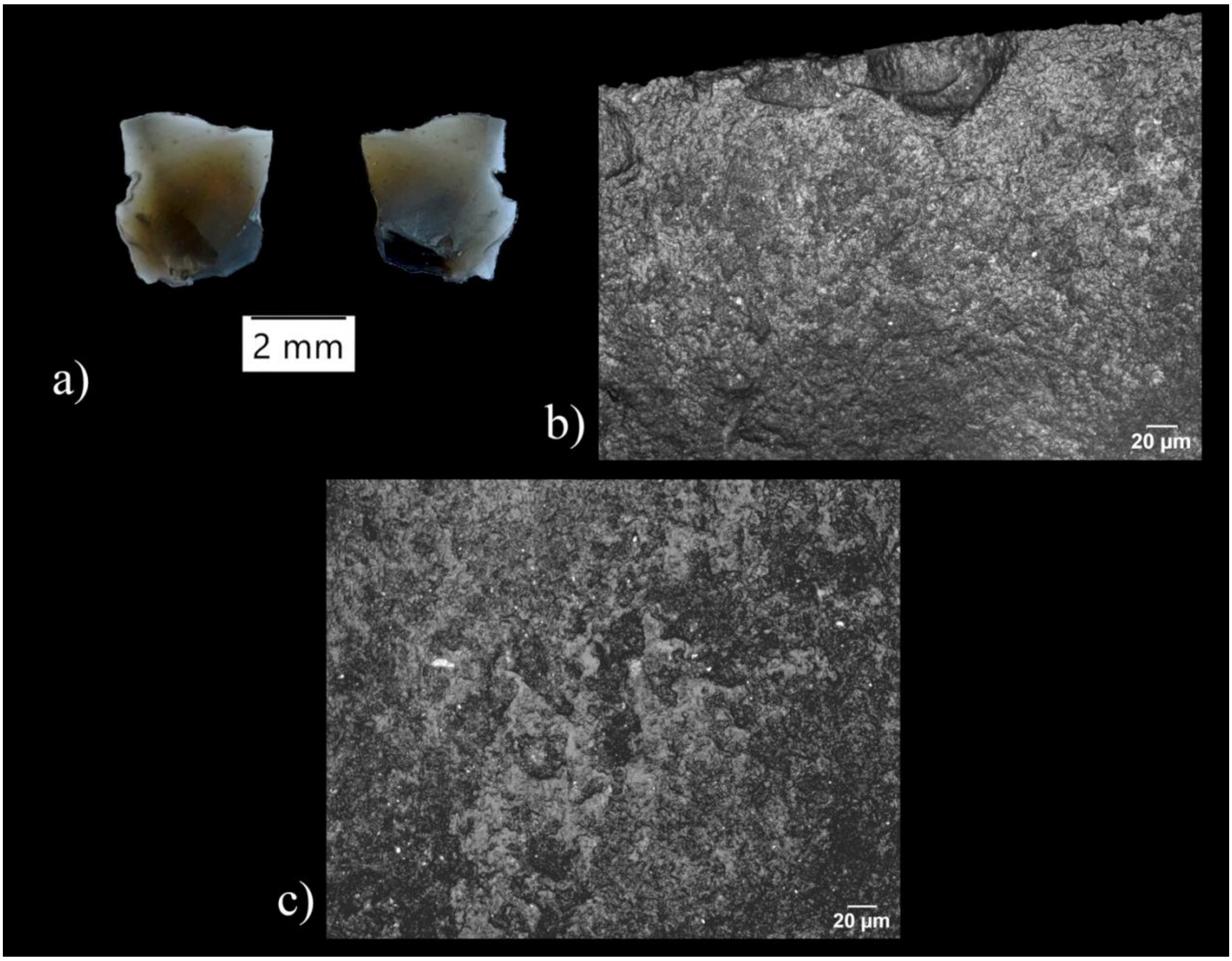

Supplementary Fig. 5. Archeological resharpening flake with use-related microwear a) microflake ID 30874; b) micropolish recorded on the dorsal platform edge (partially broken) and interpreted as working fresh wood (Magnification: 500x); c) micropolish recorded on the dorsal platform edge and interpreted as a possible superimposition between technological and use-wear traces (Magnification: 500x)

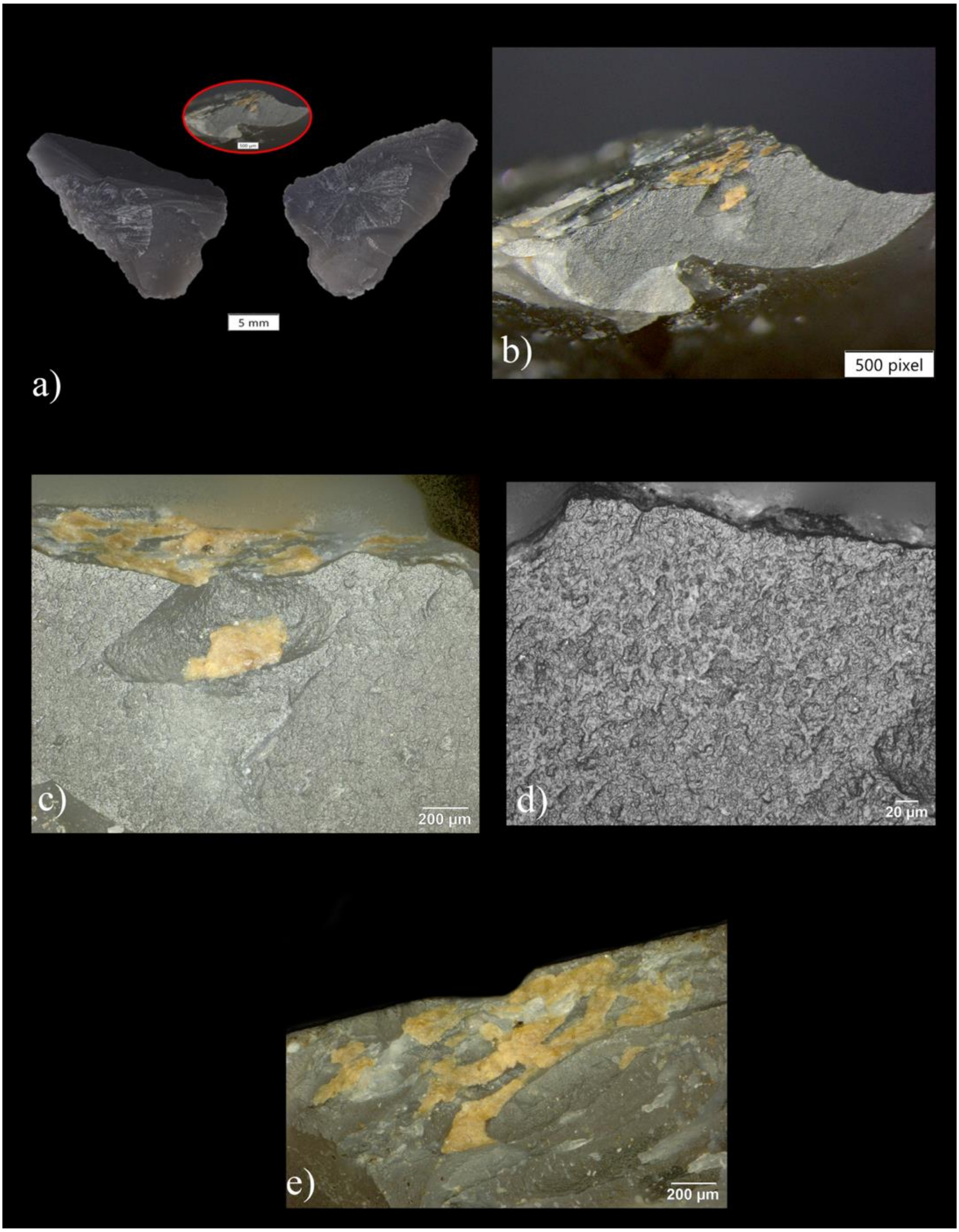

Supplementary Fig. 6. Archeological resharpening flake with use-related microwear and microresidues. a) ID 29716, notice the calcite accumulations on both faces; b) view of the butt with use-related microresidues (Magnification: 500x); c) micrograph showing the polish extension along the external platform edge (Magnification: 200x); d) micropolish recorded on the external platform edge and interpreted as working fresh wood (Magnification: 500x); e) close-up micrograph of smeared use-related residues on the retouch scars (dark field view, Magnification: 200x).

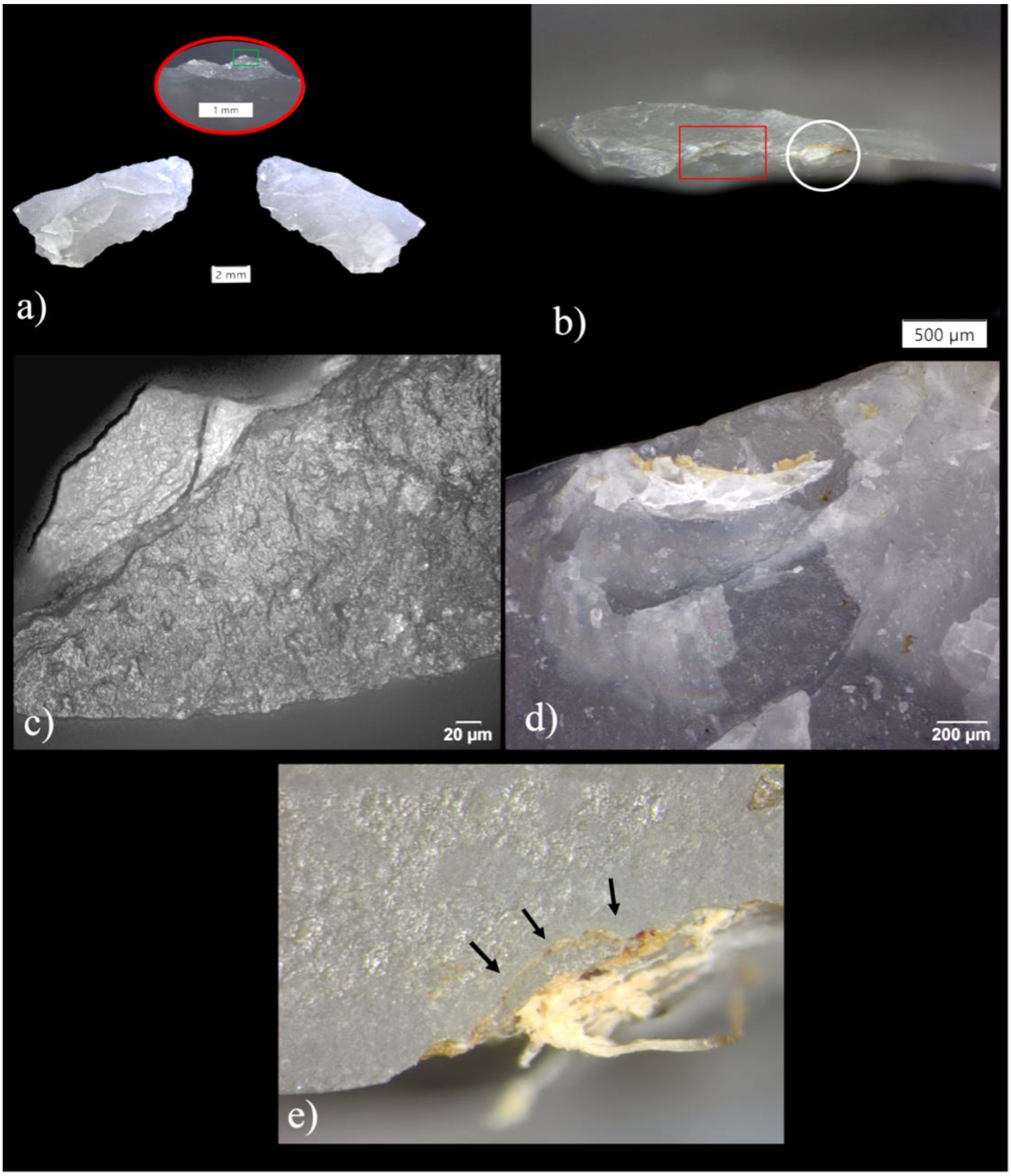

Supplementary Fig. 7. Archeological resharpening flakes with use-related use-wear and microresidues. a) microflake ID 30253 (green rectangle shows the location of micrograph (c); b) view of the butt, Magnification: 56x (the white circle indicates the location of microresidues entrapped on a fissure created at the interface between the resharpening flake and a non-detached chip while red rectangle shows the location of micrograph (d); c) weak micropolish interpreted as not sufficiently diagnostic (Magnification: 500x); d) microresidues stuck on the dorsal proximal retouch scars (Magnification: 200x); e) experimental woody microresidues entrapped on a fissure created at the interface between the resharpening flake and a non-detached chip.

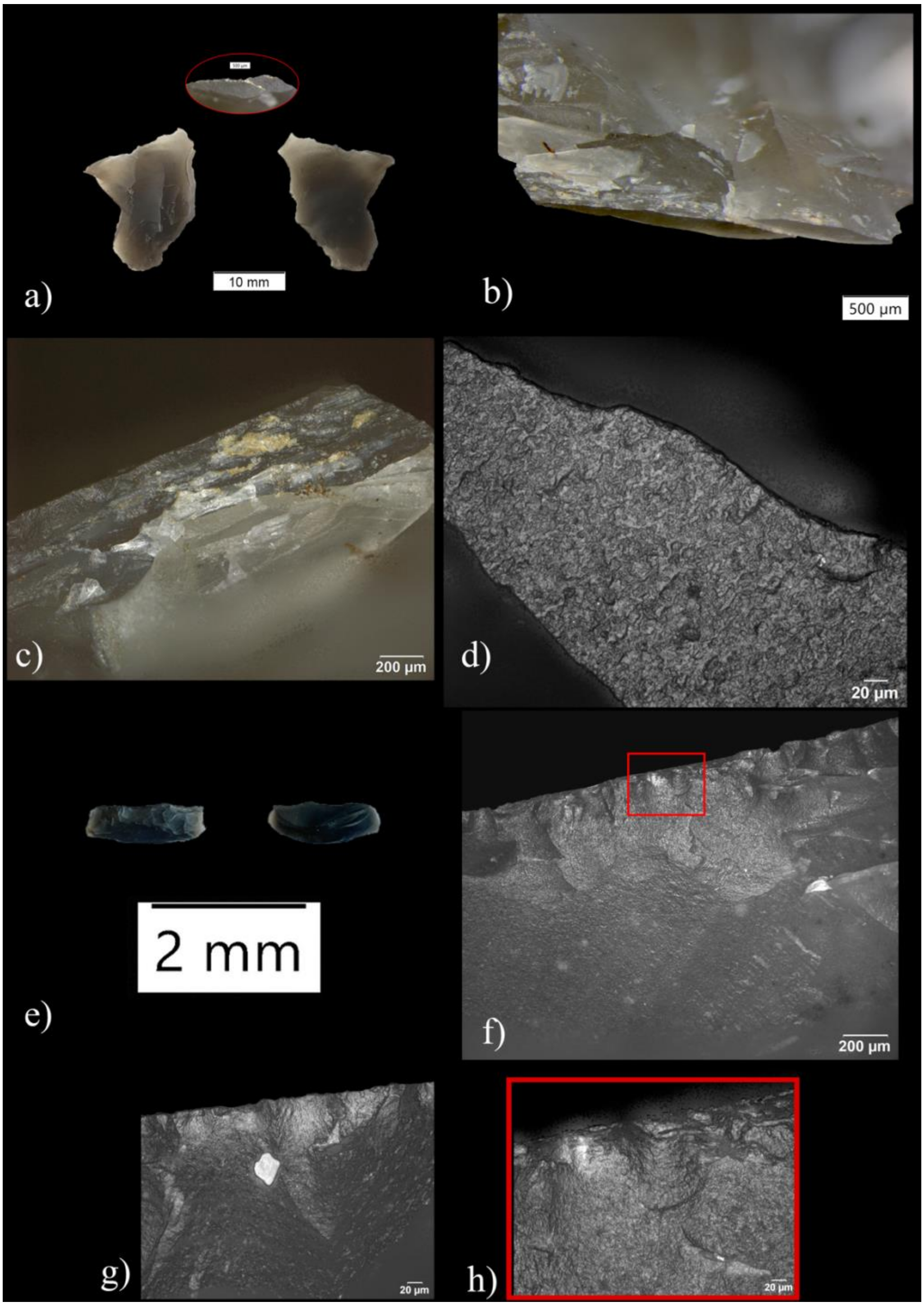

Supplementary Fig. 8. Archeological resharpening flakes with use-related use-wear and microresidues. a) Microflake ID 28802, b) view of the dorsal retouch scars with use-related microresidues (Magnification: 500x); c) close-up of the entrapped use-related microresidues (Magnification: 200x); d) weak micropolish interpreted as not sufficiently diagnostic (Magnification: 500x); e) Microflake ID 29711, f) edge damage along the dorsal edge (Magnification: 200x, red rectangle shows the close up in (h); g) wood-like polish developed inside the retouch scars along the dorsal proximal edge (Manification: 500x); h) close-up of the wood-like polish and edge rounding developed along the dorsal proximal edge (Manification: 500x).

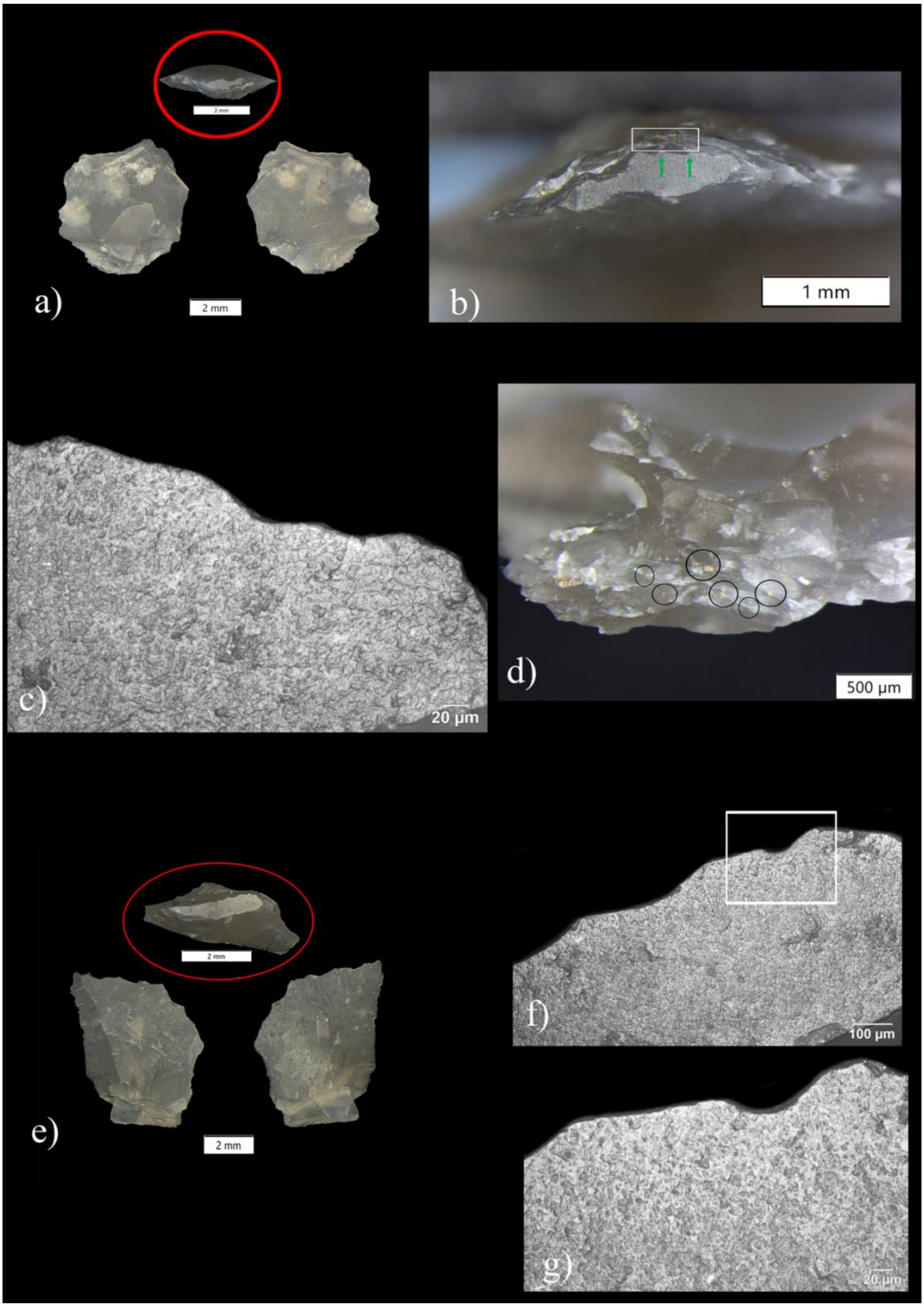

Supplementary Fig. 9. Archeological resharpening flakes with use-related use-wear and microresidues. a) Microflake ID 30878; b) view of the butt (green arrows indicate the external platform edge and the white square indicates the location of microresidues; (Magnification: 40x) c) microwear interpreted as wood working (Magnification: 500x); d) microresidues stuck on the dorsal retouch scars (Magnification: 56x); e) microflake ID 30876; f) overview of the external platform edge (Magnification: 100x); g) close-up view of the microwear interpreted as working fresh wood (white rectangle in f, Magnification: 500x).

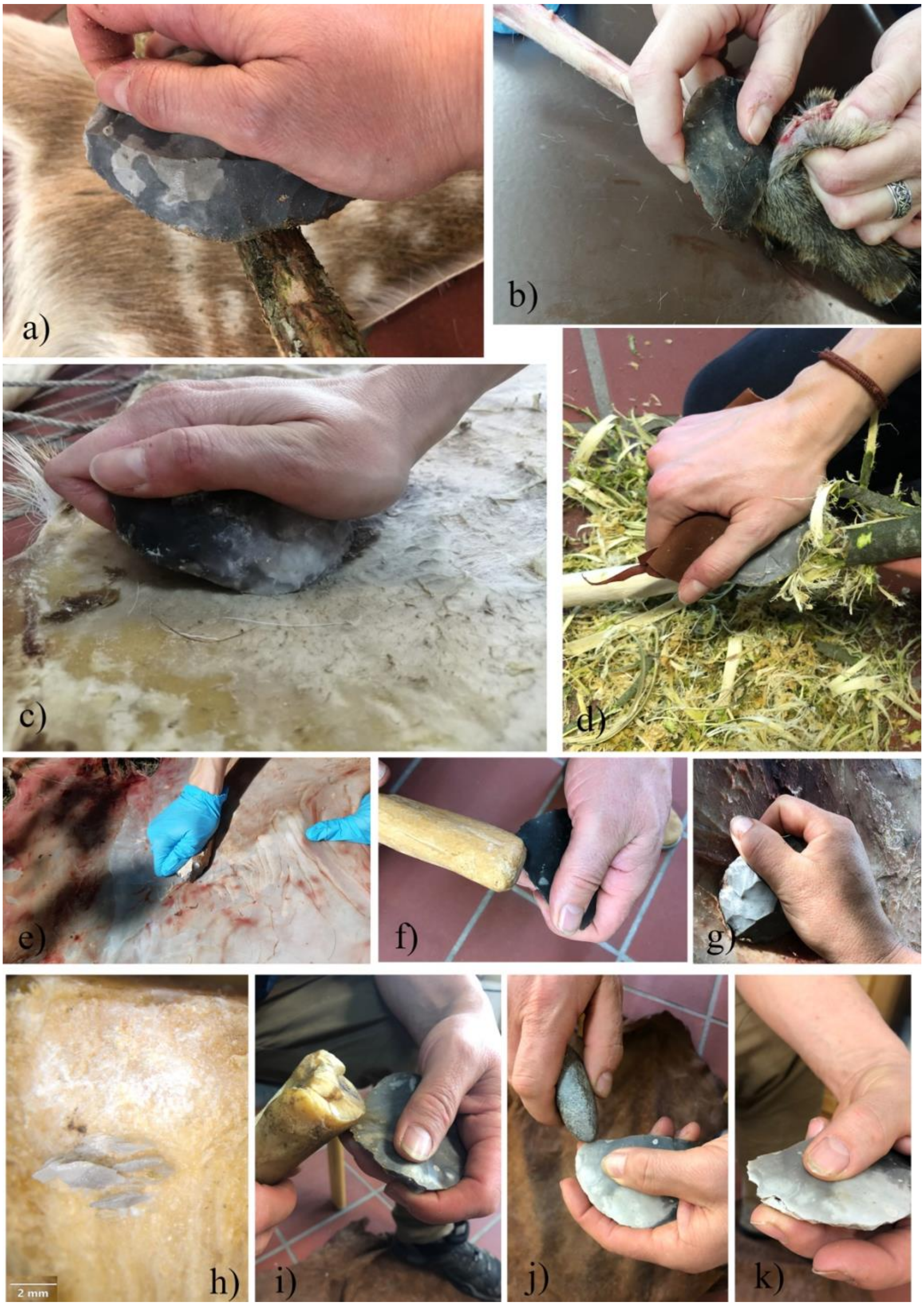

Supplementary Fig. 10. Overview of the controlled experiments: a) debarking dry wood; b) cutting animal fleshy tissues; c) scraping semi-dry hide; d) debarking fresh wood; e) scraping fresh skin; f) resharpening with boxwood; g) scraping fresh skin; h) micro flint chips embedded in the bony tissues of the experimental retoucher; i) resharpening with a bone retoucher; j) resharpening with a hammerstone; k) a detached experimental resharpening flake.

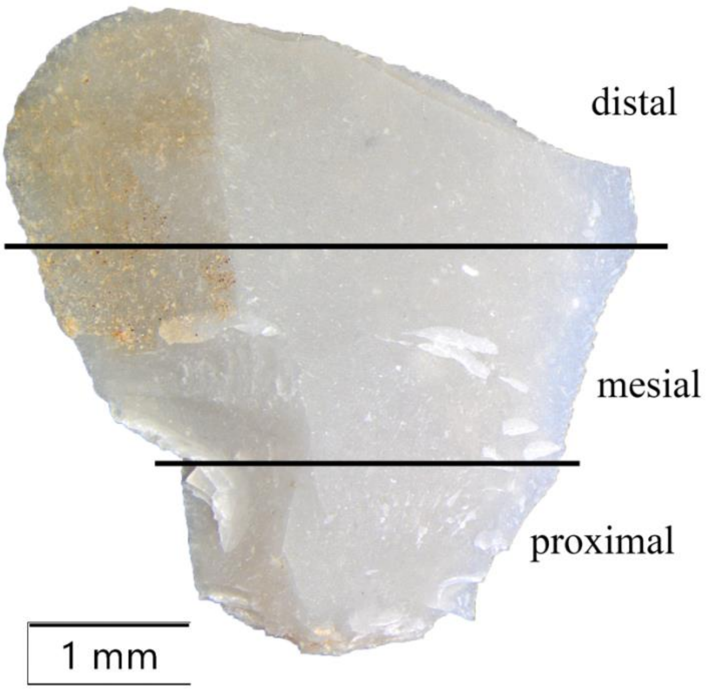

Supplementary Fig. 11. Dorsal face of an experimental resharpening flake divided in 3 portions for the spatial distribution analysis of microresidues.

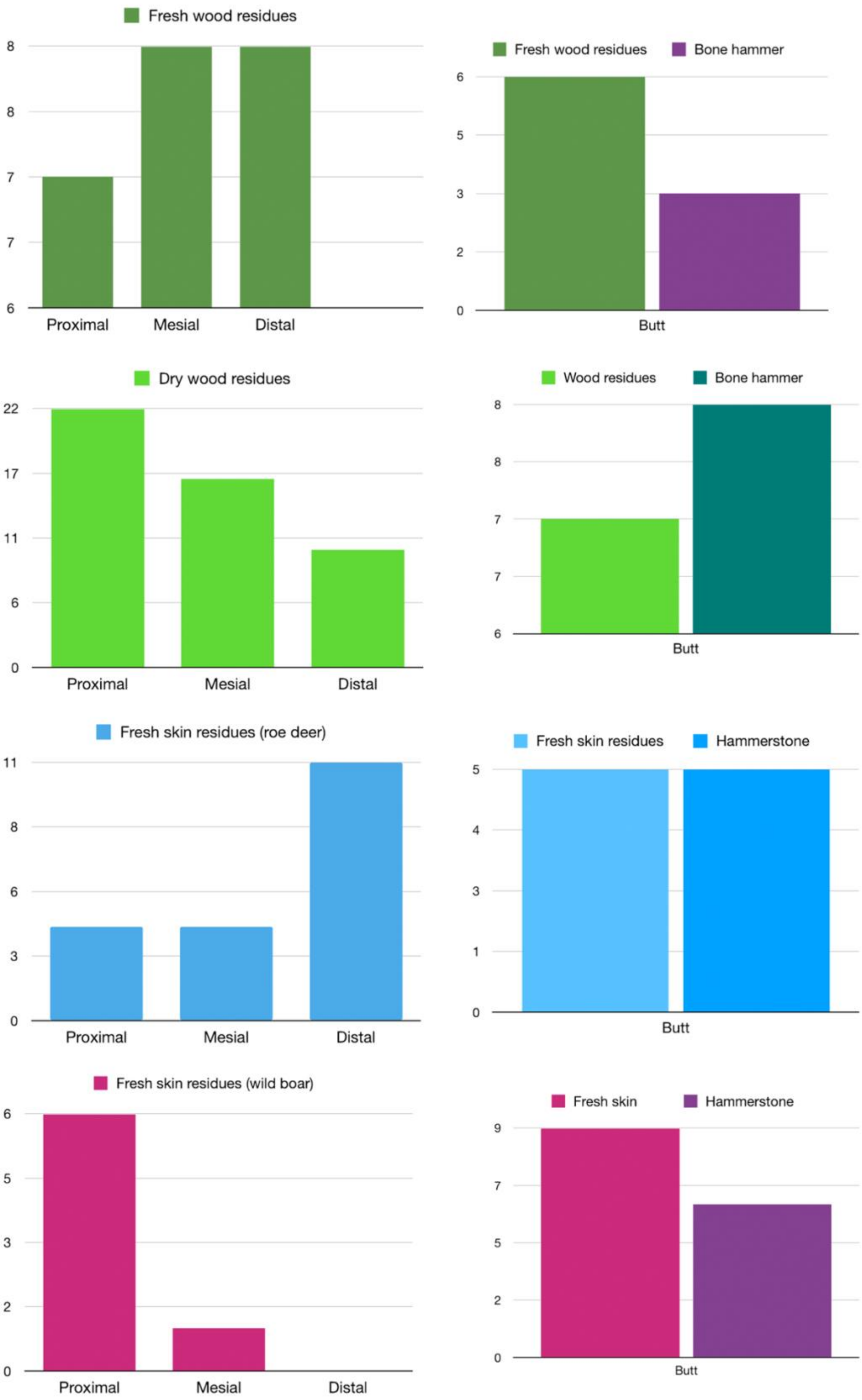

Supplementary Fig. 12. Histograms showing the pattern of distribution of microresidues on the resharpening flakes localized on the dorsal face (left) and on the butt (right).

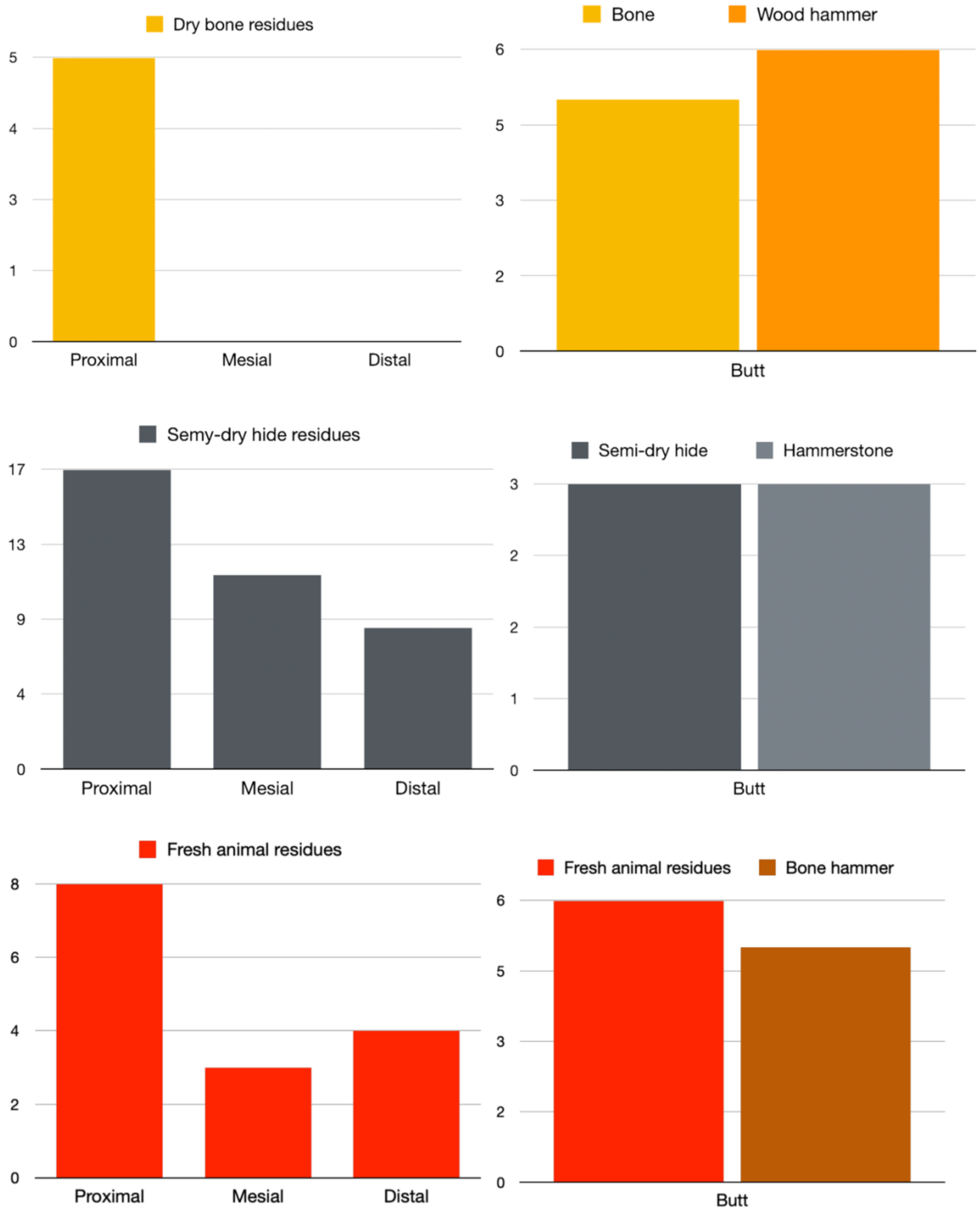

Supplementary Fig. 13. Histograms showing the pattern of distribution of microresidues on the resharpening flakes localized on the dorsal face (left) and on the butt (right).

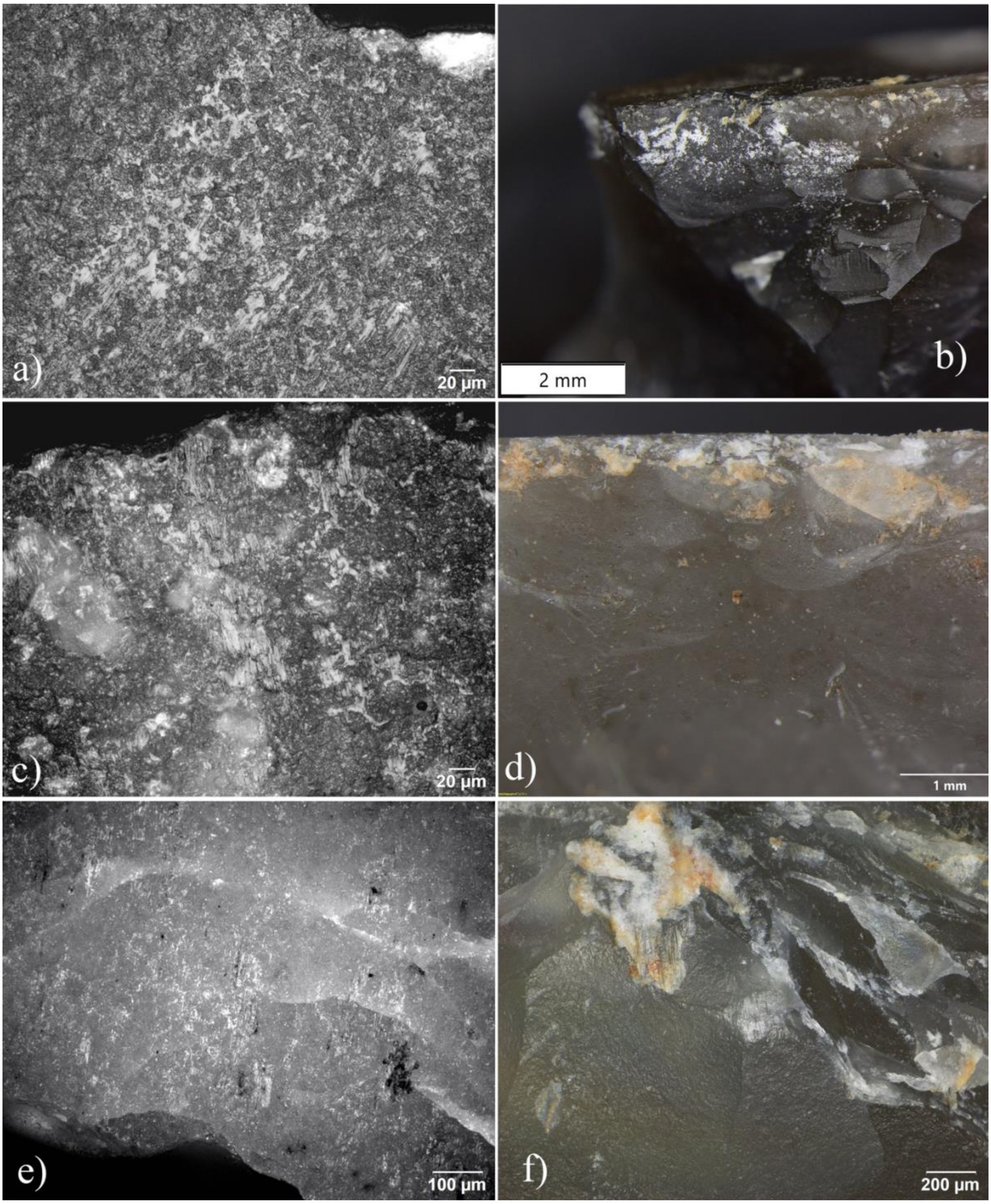

Supplementary Fig. 14. Experimental usewear and microresidues connected to resharpening. a) technological microwear produced by wood hammer (Magnification: 500x); b) overlapping of wood micro-residues along the dorsal ridge of a resharpening flake with bone use-related microresidues (Magnification: 20x); c) technological microwear produced by bone hammer (Magnification: 500x); d) overlapping of bone microresidues along the dorsal ridge of a resharpening flake with wood use-related microresidues (Magnification: 32x); e) technological microwear produced by hammerstone (Magnification: 100x); f) calcite micro-residues along the dorsal ridge of a resharpening flake (Magnification: 200x).

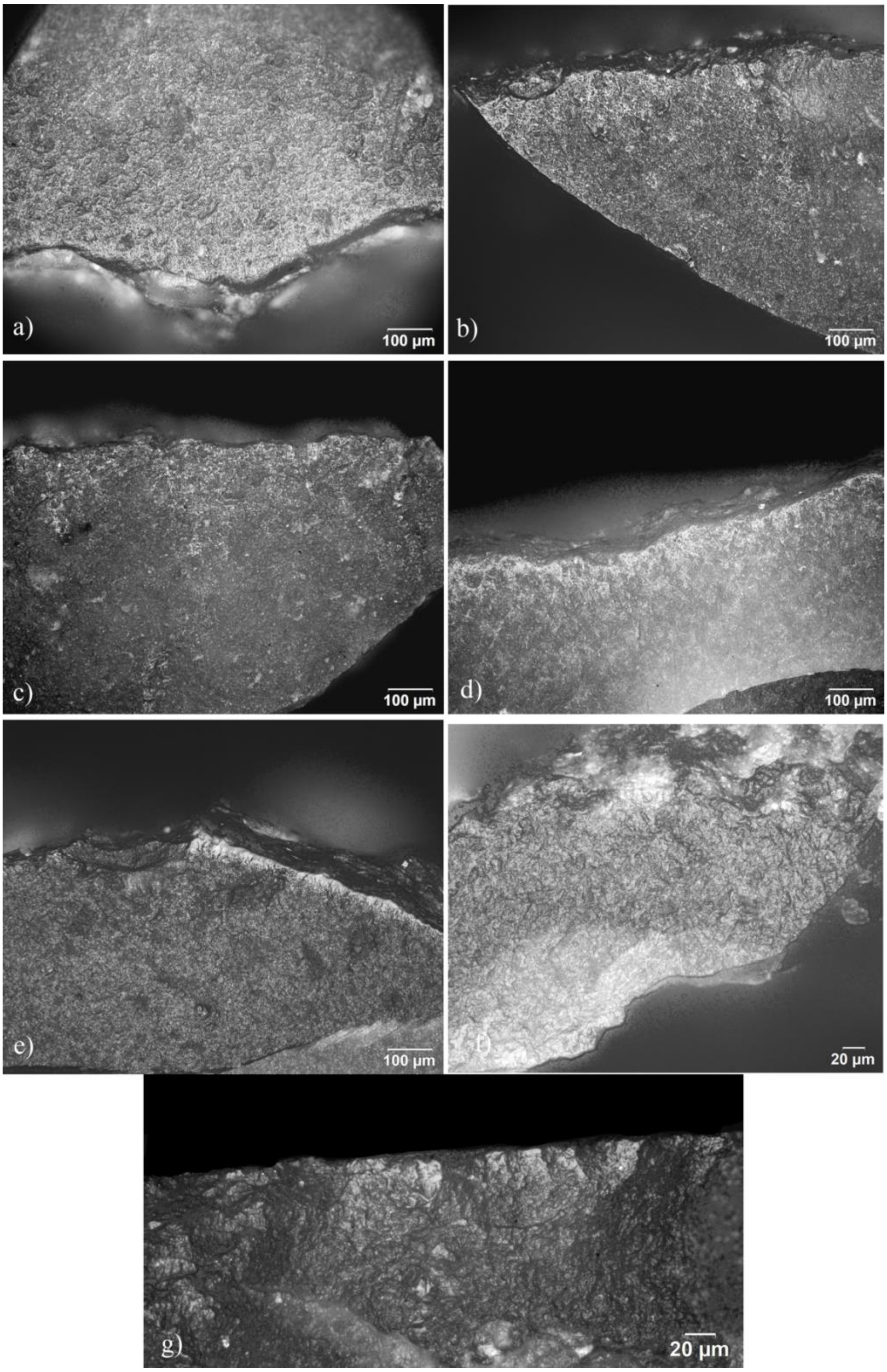

Supplementary Fig. 15. Experimental use-wear traces recorded along the external platform edge (a-f) and on the dorsal face (g) of the experimental resharpening flakes after the following activities: a) scraping fresh hide; b) butchery; c) scraping fresh wood; d) scraping semi-fresh hide; e) scraping dry bone; f) scraping fresh wood; g) scraping fresh wood. Magnification: a-e: 100x, f-g: 500x.

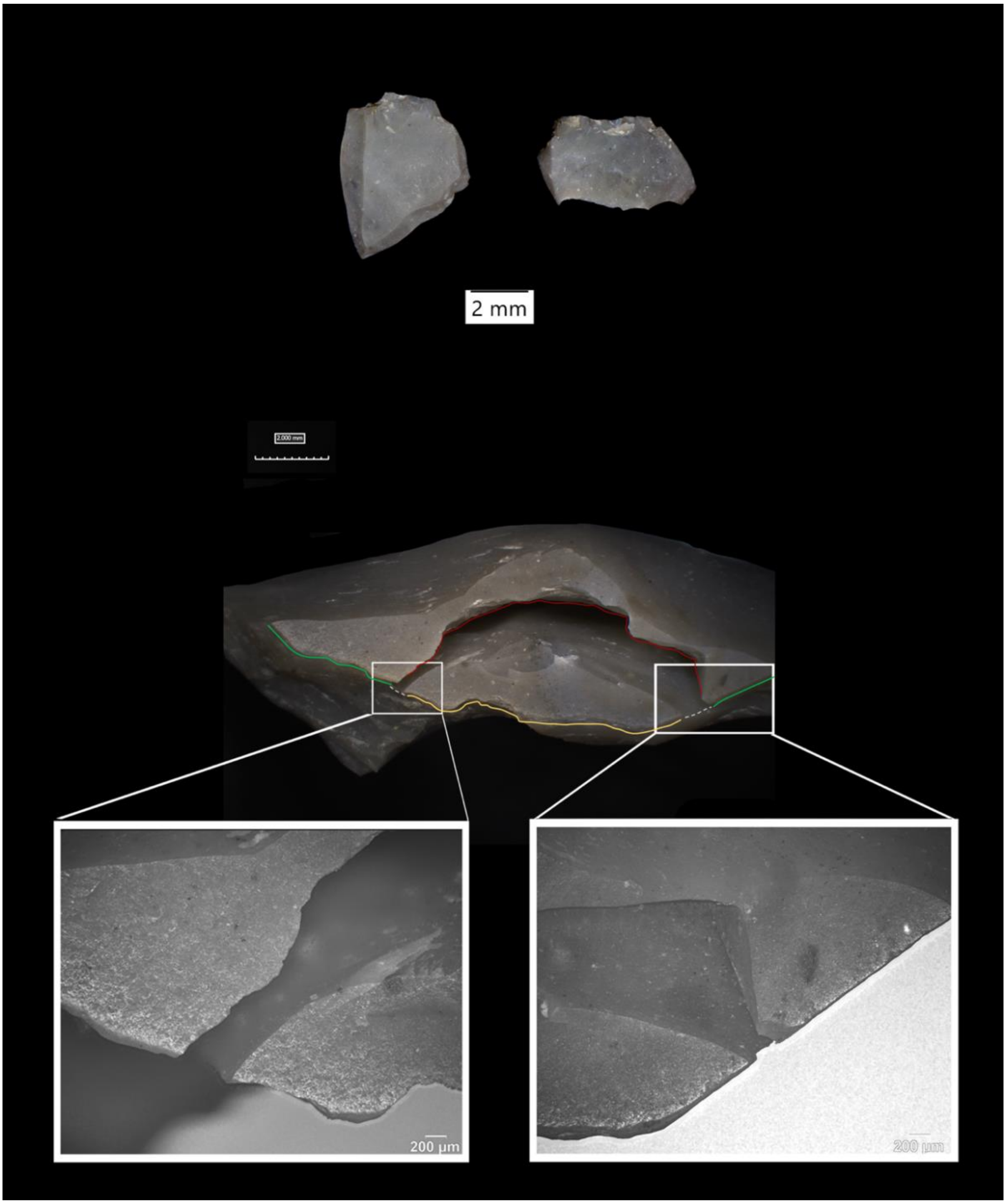

Supplementary Fig. 16. Refitting of two consecutive experimental resharpening flakes showing the distribution of the use-related microwear along the external platform edge. The yellow line represents the external platform edge corresponding to the original dorsal outer edge of the scraper, and green lines represent its continuation on the secondary detached flake. The red line indicates the continuation of the external platform edge on the secondary flake where the use-related microwear is not recorded (Magnification: 200x).

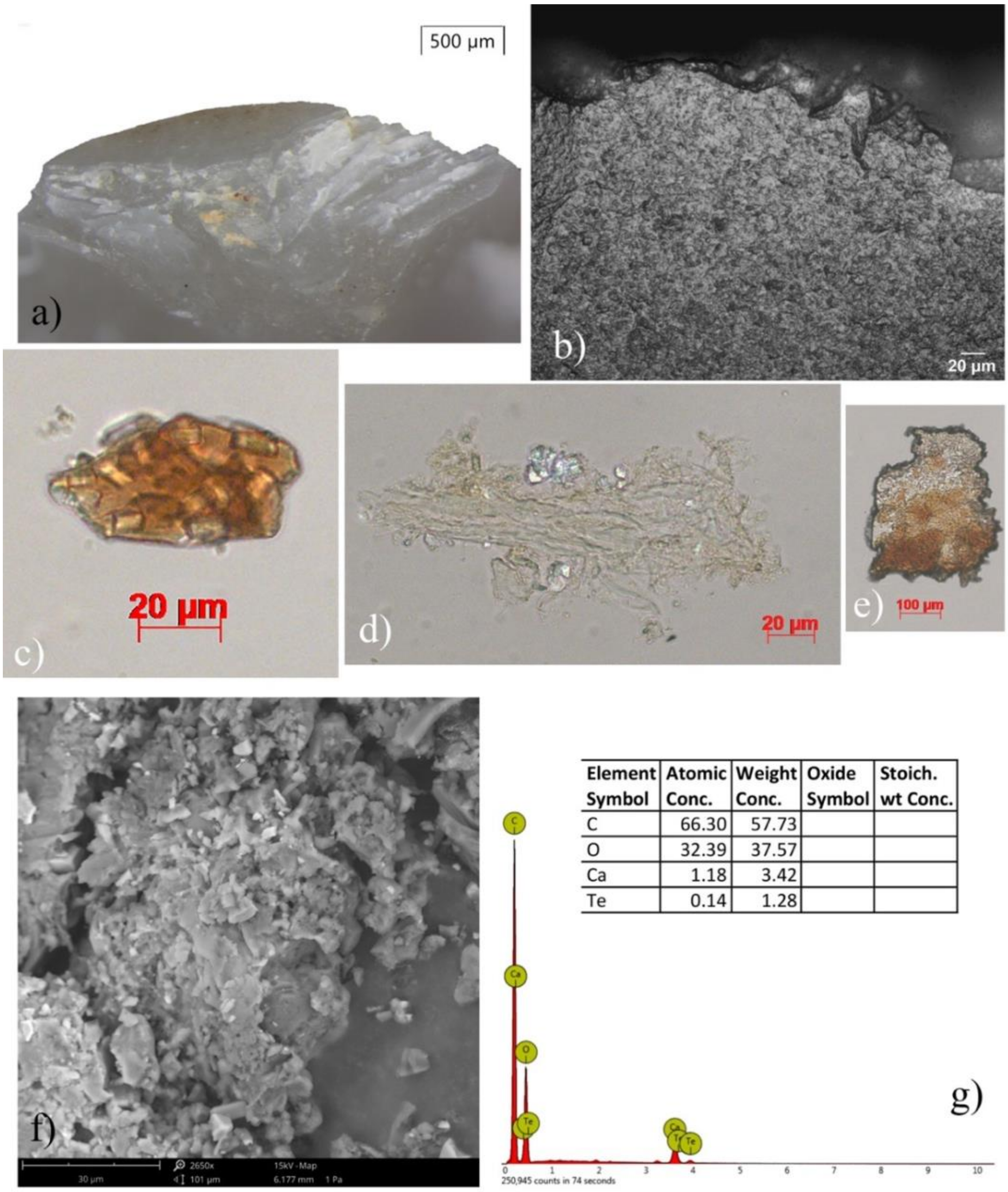

Supplementary Fig. 17. Experimental use-wear and use-related microresidues on a resharpening flake used to process wood. a) Microresidues of wood stuck on the retouch scars and smeared on the butt (Magnification: 32x); b) microwear resulting from scraping fresh spruce wood (Magnification: 500x) ; c-e) wood microresidues observed in transmitted light and polarized light (cortex and vegetal tissues, Magnification: c-d: 400x; e: 200x); f) wood microresidues imaged at the SEM; g) elemental composition of modern wood tissues.

	Complete flakes				Incomplete flakes			
	**L.**	**W.**	**Th.**	**Wg.**	**L.**	**W.**	**Th.**	**Wg.**
Max.	23	26	4	1.9	14	28	4	0.6
Min	2	2	0.5	0.05	3	4	0.5	0.05
Mean	9.0	8.3	1.3	0.2	7.9	8.7	1.3	0.1
S.D.	4.4	5.4	0.9	0.3	3.6	6.2	1.1	0.2

Supplementary Table 1. Unretouched flakes from the elephant area at Schö 13 II-3 . Maximum, minimum, mean and standard deviation (S.D.) of the length (L), width (W.), thickness (Th.) and weight (Wg.). Technological measurements. Data expressed in mm and g.

	**Complete flakes**	**Length**		**Width**		**Thickness**	
	**%**	**Mean**	**S.D.**	**Mean**	**S.D.**	**Mean**	**S.D.**
<5 mm	9.1	2.8	0.5	3.8	1.3	0.9	0.3
5 mm <10 mm	54.5	6.9	1.4	6.7	2.9	1.0	0.5
10 mm <15 mm	27.3	12.2	1.4	10.5	2.3	1.9	0.9
15 mm <20 mm	6.8	16.7	1.5	13.0	11.4	2.0	1.7
25 mm <30 mm	2.3	23	-	22	-	2	-

Supplementary Table 2. Unretouched flakes from the elephant area at Schö 13 II-3. Mean and standard deviation (S.D.) for the flaking length, width and thickness of complete products considering the flaking length intervals. Data in mm.

**Lip**		**Bulb**		**Delineation**		**Termination**	
N	30.2%	D	60.0%	ST	22.2%	F	73.5%
Y	69.8%	P	27.5%	CC	46.7%	H	20.4%
		VP	10.0%	SIN	13.3%	S	6.1%
		V	2.5%	CX	17.8%		

Supplementary Table 3. Unretouched flakes from the elephant area at Schö 13 II-3. Characteristics of the ventral surface. Lip (N: no; Y: yes); Bulb (D: diffuse, P: pronounced, VP: very pronounced, V: voluminous); Delineation (ST: straight, CC: concave, SIN: sinuous, CX: convex); Termination (F: feathered; H: hinge; S: stepped).

Corticality		Type		Preparation		Delineation	
NCO	100%	PLA	17.1%	UF	100%	ST	81.3%
		LIN	28.6%			CC	6.3%
		PLA-LIN	20.0%			SIN	12.5%
		PUN	8.6%				
		REM	25.7%				

Supplementary Table 4. Unretouched flakes from the elephant area at Schö 13 II-3. Characteristics of the butts. Corticality (NCO: non-cortical); Type (PLA: platform, LIN: linear, PUN: punctiform; REM: removed); Preparation (UF: unifaceted), Delineation (ST: straight, CC: concave, SIN: sinuous, CX: convex). Percentages of artifacts analyzed.

A

	Width	Thickness
Max.	17	3
Min.	1	0.1
Mean	3.1	0.7
S.D.	3.1	0.6

B

		Width		Thickness	
	%	Mean	S.D.	Mean	S.D.
≤1 mm	*17.2*	1	0	0.18	0.04
>1mm - 5mm	*72.4*	2.6	0.94	0.67	0.29
>5 mm - 10 mm	*6.9*	6.5	0.71	1	0
>15 mm - 20 mm	*3.4*	17	-	3	-

Supplementary Table 5. A) Maximum, minimum, mean and standard deviation (S.D.) of the width and thickness of the butts. **B**) Mean and standard deviation attending to width intervals. Percentages of artifacts analyzed from the elephant area at Schö 13 II-3.

A

Corticality		Delineation	
NCO	100%	ST	21.7 %
		CC	6.5 %
		SIN	4.3 %
		CX	47.8 %
		1A	19.6 %

B

N° of scars	
1	29.3%
2	36.6%
3	22.0%
4	9,8%
5	2.4%

C

Frontal		Sagittal		Transversal	
TRG	7.5%	TRG	81.4%	TRG	86.4%
PLG	27.5%	S/OV	4.7%	S/OV	6.8%
TRP	40.0%	TRP	14.0%	TRP	6.8%
S/OV	22.5%				
QDG	2.5%				

Supplementary Table 6.
Unretouched flakes from the elephant area at Schö 13 II-3. **A)** Characteristics of the dorsal surface. Corticality of complete flakes (NCO: non-cortical); Delineation (ST: straight, CC: concave; SIN: sinuous, CX: convex, 1A: angular). **B)** Number of dorsal scars and **C)** morphologies: frontal view (just complete flakes), vertical and horizontal sections (TRG: triangular, PLG: polygonal, TRP: trapezoidal, S/OV: semi oval/oval; PTG: pentagonal, QDG: quadrangular). Percentages of artifacts analyzed.

ID Flake	Use-wear traces	Use-related residues	Residues analysis	Overall Interpretation
30876	**Edge rounding:** Medium **Edge damage**: Feather **Polish Direction and Texture**: Transversal, Rough **Polish Topography**: Granular + Wet appearance	No residues identified	/	Woodworking, transversal motion
29716	**Edge rounding:** Medium **Edge damage**: / **Polish Direction and Texture**: Slightly oblique, Rough **Polish Topography**: Granular + Wet appearance	Dense amorphous yellowish-orange residues with brownish shades firmly adhered on the striking platform and smeared in the dorsal proximal retouch scars **Location**: dorsal proximal retouch scars + butt	Optical observations in reflected light<br><br>FTIR on residues: Not diagnostic)	Woodworking, transversal motion
30878	**Edge Rounding:** Low **Edge damage**: / **Polish Direction and Texture**: Transversal, Rough **Polish Topography**: Granular + Wet appearance	Amorphous yellowish-orange small residues stuck in the dorsal proximal retouch scars **Location**: dorsal proximal retouch scars	Optical observations in reflected light	Woodworking, transversal motion
30873	**Edge Rounding:** Medium **Edge damage**: / **Polish Direction and Texture**: / **Polish Topography**: /	Amorphous yellowish-orange small residues stuck in the dorsal proximal retouch scars **Location**: dorsal proximal retouch scars	Optical observations in reflected light	Woodworking
30251	**Edge Rounding:** Medium **Edge damage**: Little feather **Polish Direction and Texture**: Weakly developed, slightly oblique, rough **Polish Topography**: Granular + Wet appearance	Dense amorphous yellowish-orange residues with brownish shades firmly adhered in the dorsal proximal retouch scars **Location**: dorsal proximal retouch scars + butt	• FTIR on residues (Not diagnostic)<br>• SEM-EDX: wood particles<br>• Optical observations in reflected and transmitted light	Woodworking, transversal motion
30874	**Edge Rounding:** Medium **Edge damage**: Hinge **Polish Direction and Texture**: Transversal, Rough **Polish Topography**: Granular + Wet appearance	No residues identified	/	Woodworking, transversal motion
30745	**Edge Rounding:** Medium **Edge damage**: / **Polish Direction and Texture**: Transversal, Rough **Polish Topography**: Granular + Wet appearance	Amorphous yellowish-orange small residues stuck in the dorsal proximal retouch scars **Location**: dorsal proximal retouch scars	Optical observations in reflected light	Woodworking

30495	**Edge Rounding:** Medium **Edge damage**: Feather **Polish Direction and Texture**: Transversal, Rough **Polish Topography**: Granular	No residues identified	/	Woodworking
29632	**Edge Rounding: /** **Edge damage**: / **Polish Direction and Texture**: Transversal, Rough to Smooth **Polish Topography**: Domed	No residues identified	/	Woodworking
29711	**Edge Rounding:** Low **Edge damage**: Feather + Hinge **Polish Direction and Texture**: Transversal, Rough **Polish Topography**: Granular + Wet appearance	No residues identified	/	Woodworking
29804	**Edge Rounding: /** **Edge damage**: / **Polish Direction and Texture**: Transversal, Rough **Polish Topography**: Granular + Wet appearance	Amorphous yellowish-orange small residues adhering to the dorsal proximal retouch scars **Location**: dorsal proximal retouch scars	Optical observations in reflected light	Woodworking
30253	Extremely weak polish, indeterminable	Amorphous yellowish-orange small residues adhering to the dorsal proximal retouch scars and entrapped in a non-detached chip **Location**: dorsal proximal retouch scars	Optical observations in reflected light	Woodworking
30492	**Edge Rounding:** Low **Edge damage**: Feather + Hinge **Polish Direction and Texture**: Transversal, Rough **Polish Topography**: Flat/Domed + Wet appearance	No residues identified	/	Woodworking
30255	Extremely weak polish, indeterminable	Amorphous yellowish-orange residues with brownish shades firmly adhered on the striking platform and smeared in the dorsal proximal retouch scars. **Location**: dorsal proximal retouch scars	Optical observations in reflected light<br><br>FTIR on sediment	Woodworking
28802	**Edge Rounding:** High **Edge damage**: Feather **Polish Direction and Texture**: Weakly developed, transversal, rough **Polish Topography**: Granular + Wet appearance	Amorphous yellowish-orange residues with brownish shades firmly adhered on the striking platform and smeared in the dorsal proximal retouch scars. **Location:** dorsal proximal retouch scars + butt	Optical observations in reflected light	Woodworking

29820	Extremely weak polish, indeterminable	Amorphous yellowish-orange residues with brownish shades firmly adhered on the striking platform and smeared in the dorsal proximal retouch scars. **Location**: dorsal proximal retouch scars + butt	Optical observations in reflected light	Woodworking
28771	**Edge Rounding:** Medium **Edge damage**: / **Polish Direction and Texture**: Transversal, Rough **Polish Topography**: Granular + Wet appearance	Amorphous yellowish-orange small residues stuck in the dorsal proximal retouch scars. **Location**: dorsal proximal retouch scars	Optical observations in reflected light	Woodworking
30279	**Edge rounding:** / **Edge damage**: / **Polish Texture**: Rough **Polish Topography**: Granular + Wet appearance	Amorphous yellowish-orange small residues stuck in the dorsal proximal retouch scars. **Location**: dorsal proximal retouch scars	Optical observations in reflected light	Woodworking
12.9.18 A	**Edge rounding:** Medium **Edge damage**: feather **Polish Texture**: Rough **Polish Topography**: Granular + Wet appearance	Amorphous yellowish-orange residues with brownish shades firmly adhered on the striking platform. **Location**: butt	Optical observations in reflected light	Woodworking
30493	Extremely weak polish, indeterminable	No residues identified	/	Indeterminable/ Generic contact
29899	Extremely weak polish, indeterminable	No residues identified	/	Indeterminable/ Generic contact

Supplementary Table 7: Comparative table of use-wear and residues identified on the archeological resharpening flakes.

Number	ID replica	Edge angle	Contact material	Type of contact material	Action	Contact angle	Total time *min*	Hammer	Amount of produced microdebitage ≥ 2 mm
1	Scraper #01	70°	Fresh wood	*Salix*	Scraping/ debarking	45°	60	Bone	24 resharpening flakes
2	Scraper #03	84°	Dry wood	*Picea* sp.	Scraping/ debarking	45°	60	Bone	27 resharpening flakes
3	Scraper #08	92°	Fresh skin	*Cervus capreolus*	Scraping	45°	110	Stone	21 resharpening flakes
4	Scraper #07	53°	Fresh skin	*Sus scrofa*	Scraping	45°+90°	60	Stone	16 resharpening flakes
5	Scraper #04	58°	Semi-dry hide	*Cervus elaphus*	Scraping	45°	60	Stone	25 resharpening flakes
6	Scraper #02	54°	Dry bone	*Cervus capreolus*	Scraping	45°	60	Wood	19 resharpening flakes
7	Scraper #05	62°	Butchery	*Ovis gmelini*	Multiple actions (cutting uni- and bidirectional + scraping)	90°+45°	30	Bone	20 resharpening flakes

Supplementary Table 8. Experimental variables recorded for the seven scrapers used during the experimental trials.
